# Comparative transcriptome and translatome analysis in contrasting rice genotypes reveals differential mRNA translation in salt-tolerant Pokkali under salt stress

**DOI:** 10.1186/s12864-018-5279-4

**Published:** 2018-12-31

**Authors:** Yong-Fang Li, Yun Zheng, Lakshminarayana R. Vemireddy, Sanjib Kumar Panda, Smitha Jose, Alok Ranjan, Piyalee Panda, Ganesan Govindan, Junxia Cui, Kangning Wei, Mahmoud W. Yaish, Gnanambal Charmaine Naidoo, Ramanjulu Sunkar

**Affiliations:** 1 College of Life Sciences, Henan Normal University, Xinxiang, 453007 Henan China; 2 Department of Biochemistry and Molecular Biology, Oklahoma State University, Stillwater, OK 74078 USA; 3 Yunnan Key Lab of Primate Biomedicine Research; Institute of Primate Translational Medicine, Kunming University of Science and Technology, Kunming, Yunnan 650500 China; 4 Department of Biology, College of Science, Sultan Qaboos University, Muscat, Oman; 5 Department of Biology, Langston University, Langston, OK 73050 USA

**Keywords:** Gene regulation, Polysomal RNA-Seq, Rice, RNA-Seq, Salt stress, Transcription, Translation

## Abstract

**Background:**

Soil salinity is one of the primary causes of yield decline in rice. Pokkali (Pok) is a highly salt-tolerant landrace, whereas IR29 is a salt-sensitive but widely cultivated genotype. Comparative analysis of these genotypes may offer a better understanding of the salinity tolerance mechanisms in rice. Although most stress-responsive genes are regulated at the transcriptional level, in many cases, changes at the transcriptional level are not always accompanied with the changes in protein abundance, which suggests that the transcriptome needs to be studied in conjunction with the proteome to link the phenotype of stress tolerance or sensitivity. Published reports have largely underscored the importance of transcriptional regulation during salt stress in these genotypes, but the regulation at the translational level has been rarely studied. Using RNA-Seq, we simultaneously analyzed the transcriptome and translatome from control and salt-exposed Pok and IR29 seedlings to unravel molecular insights into gene regulatory mechanisms that differ between these genotypes.

**Results:**

Clear differences were evident at both transcriptional and translational levels between the two genotypes even under the control condition. In response to salt stress, 57 differentially expressed genes (DEGs) were commonly upregulated at both transcriptional and translational levels in both genotypes; the overall number of up/downregulated DEGs in IR29 was comparable at both transcriptional and translational levels, whereas in Pok, the number of upregulated DEGs was considerably higher at the translational level (544 DEGs) than at the transcriptional level (219 DEGs); in contrast, the number of downregulated DEGs (58) was significantly less at the translational level than at the transcriptional level (397 DEGs). These results imply that Pok stabilizes mRNAs and also efficiently loads mRNAs onto polysomes for translation during salt stress.

**Conclusion:**

Under salt stress, Pok is more efficient in maintaining cell wall integrity, detoxifying reactive oxygen species (ROS), translocating molecules and maintaining photosynthesis. The present study confirmed the known salt stress-associated genes and also identified a number of putative new salt-responsive genes. Most importantly, the study revealed that the translational regulation under salinity plays an important role in salt-tolerant Pok, but such regulation was less evident in the salt-sensitive IR29.

**Electronic supplementary material:**

The online version of this article (10.1186/s12864-018-5279-4) contains supplementary material, which is available to authorized users.

## Background

Soil salinity is one of the primary causes of yield decline in rice. An estimated 20% of cultivated land and 33% of irrigated agricultural land are afflicted by high salinity [1]. Furthermore, soil salinity has increased at an alarming rate worldwide, including in south and southeast Asian countries, where rice is widely grown. Salinity has a negative effect on crop production: even moderate salt stress can reduce crop yield substantially. The adverse effects of salinity on plants include Na^+^ toxicity, osmotic and oxidative stresses as well as imbalance in ionic homeostasis, particularly K^+^ and Ca^2+^ [2–4]. Understanding the molecular basis of salt tolerance is a prerequisite for developing salt-tolerant rice varieties. Rice is endowed with rich genetic diversity [5]. Among diverse salt-tolerant rice genotypes, Pok is widely used as one of the best genotypes for dissecting salt tolerance in rice. On the other hand, IR29 is a modern, high-yielding cultivar but highly salt-sensitive genotype [6, 7]. Comparative analysis of these rice genotypes may lead to a better understanding of the salt tolerance mechanism at the molecular level.

The regulation of gene expression in a cell operates at epigenomic, transcriptional, translational and post-translational levels [8]. Although most stress-responsive genes are regulated at the transcriptional level, in many cases changes at this level are not often reflected with a change in protein abundance, which suggests that transcriptional regulation does not always account for altered protein accumulation [8–13]. These observations also suggest that analysis of proteomic changes in addition to the transcriptome under stress is almost indispensable to link the phenotype with stress tolerance or sensitivity. Mass spectrometry-based analyses of the proteome is widely used to measure protein expression at the global scale. Alternatively, the translatome (mRNAs associated with polysomal fractions) could mimic the proteome component in cells [14–17]. Furthermore, high throughput sequencing of the translatome can identify genes with low expression that might be missed by proteomic analysis [18].

Previous studies have apllied microarray analysis to identify differentially expressed genes (DEGs) in Pok and IR29 genotypes exposed to salt stress [19–22]. However, the sensitivity of microarray-based profiling is low for identifying genes with low expression and yet differentially responding to stress. RNA-Seq is highly sensitive and can identify genes that are low-abundantly expressed as well as novel genes, besides distinguishing the alternatively spliced variants. Besides the transcriptome, the analyses of proteome component is critically important to understand overall changes in protein regulation during stress. In this study, we used RNA-Seq to analyze transcriptome as well as translatome (mimicking the proteome component) of Pok and IR29 under salt stress. The transcriptome and translatome profiles revealed distinct differences between Pok and IR29 without salt stress (basal expression). Importantly, under salt stress, Pok seemed to stabilize mRNAs of the stress-adaptive genes and also efficiently load mRNAs onto the polysomes for translation. The overrepresented Gene Ontology (GO) terms for DEGs of Pok compared to IR29 include maintaining cell wall integrity and photosynthesis, translocating molecules, and detoxifying ROS under salt stress.

## Results and discussion

### A comprehensive view of the sequenced transcriptomes and translatomes

**Fig. 1:**
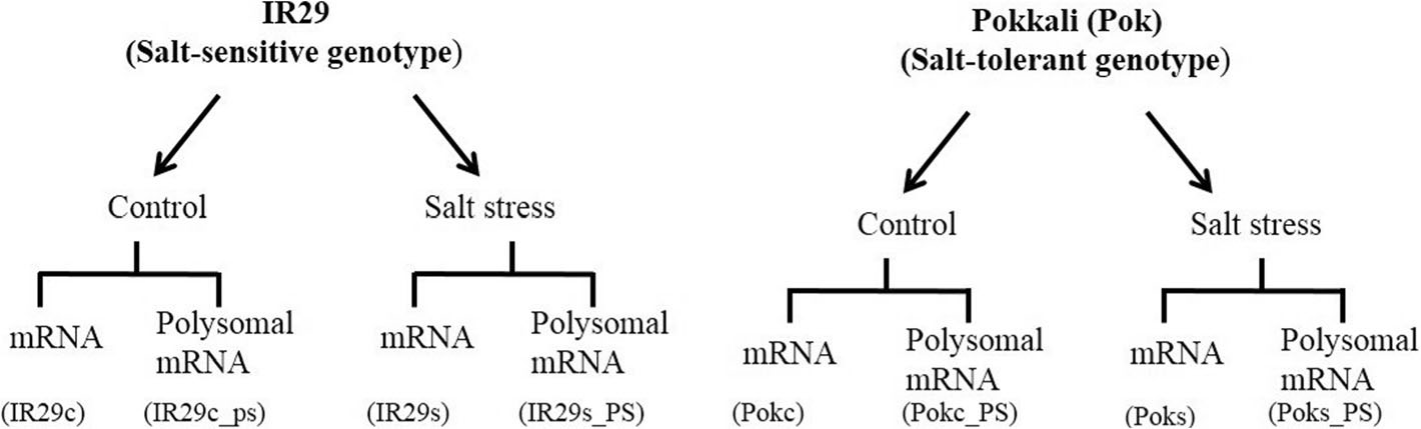
Schematic representation of RNA-Seq libraries sequenced from IR29 (salt-sensitive genotype) and Pokkali (Pok; salt-tolerant genotype) under control and salt stress conditions

**Fig. 2:**
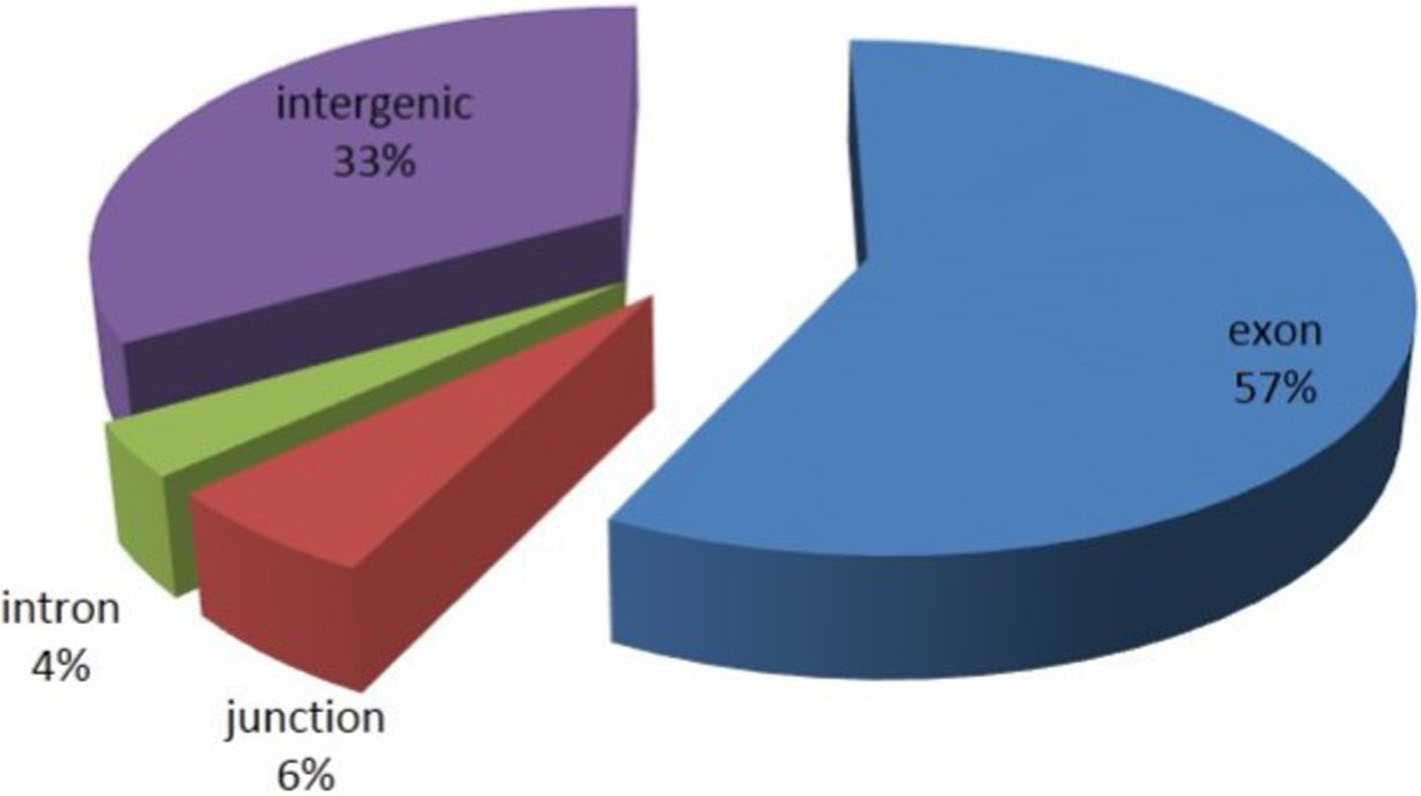
Distribution of filtered reads in different categories (exon, intron, junction and intergenic regions)

Comparative transcriptome (mRNA) and translatome (RNA associated with polysomes [PS-mRNA]) profiles in salt-tolerant Pok and salt-sensitive IR29 genotypes should elucidate transcriptional and translational differences between these contrasting genotypes. To profile gene expression changes, 24-day-old Pok and IR29 seedlings grown on Yoshida medium were exposed to 150 mM NaCl for 24 h or continued to grow on Yoshida medium as a control. Total mRNA and mRNA associated with the polysomes were isolated from the control and salt-treated seedlings and used for constructing RNA-Seq libraries. We generated eight RNA-Seq libraries, four libraries each for Pok and IR29 (mRNA and mRNA-associated with the polysomes from control and salt-treated samples for each genotype) (Fig. 1). Upon sequencing these libraries by using the single-end Illumina platform, 937,066,680 reads of 50- to 51-nt long were obtained. The sequencing depth ranged from 67 to 170 million reads for each library. The raw sequences were inspected, and low-quality reads were discarded. The remaining 731,604,828 reads (78% of total raw reads) that ranged from 58 to 121 million reads for each library (Table 1) were mapped to the rice genome (*Oryza sativa subsp. japonica;* MSU genome and transcriptome annotations – v6.1). They covered about 77% of the annotated genes of the genome. Most of the uniquely mapped reads were aligned to the exons (57%), followed by intergenic regions (33%), whereas a small fraction was mapped to splice junctions (6%) and introns (4%) (Fig. 2). Mapping of a very high proportion of the reads to exonic regions indicates the high quality of RNA-Seq libraries.

**Table 1:** Summary of RNA-Seq and Polysomal RNA-seq reads sequenced from control and salt-stressed Pok and IR29

	IR29c_RNA	IR29s_RNA	POKc_RNA	POKs_RNA	IR29c_PS	IR29s_PS	POKc_PS	POKs_PS
Raw reads	96,100,081	85,472,491	101,585,264	67,036,814	170,629,357	157,045,640	148,401,455	110,795,578
Filtered reads	78,181,515	70,361,944	83,082,414	58,994,184	121,305,093	119,567,766	116,502,326	83,609,586
cDNA	64,774,078	56,675,885	69,259,043	46,680,420	104,283,746	80,032,608	54,405,711	47,522,099
Exons	58,116,153	50,855,521	63,980,331	43,862,264	94,258,549	71,885,574	49,107,464	43,386,281
Junctions	6,657,925	5,820,364	5,278,712	2,818,156	10,025,197	8,147,034	5,298,247	4,135,818
Introns	3,444,625	2,926,711	6,002,251	3,081,373	4,601,871	4,001,816	2,295,597	1,813,751
Intergenic regions	30,074,168	25,475,468	43,686,405	31,329,140	55,664,740	38,593,396	27,603,916	24,192,773

IR29c RNA and IR29s denotes the RNA-Seq libraries of IR29 under control and salt stress conditions, respectively. The columns ending with PS denotes the polysomal RNA-Seq libraries (translatome)

After mapping the sequenced reads to the rice genome, 89,715 transcripts were assembled from the eight sequencing libraries; 64.2% belonged to the annotated genes, 22.4% to the alternatively spliced transcripts, and 3.5% to the novel transcripts in intergenic regions (Additional file 1: Table S1). Identifying significant percenetage of alternatively spliced transcripts suggests that the annotated genes may have a sizable number of alternatively spliced gene products. To quantify the differences in gene expression profiles in response to salt stress, the gene expression level was normalized by using Fragments Per Kilobase of transcripts per Million mapped reads (FPKM). We exclude the assembled transcripts of < 5 FPKM in all eight libraries from further analyses and the remaining 12,622 annotated transcripts were clustered by subjecting them to *K*-Means Clustering by using Euclidian distance measures with Genesis v1.8.1 [23]. The results revealed a distinct expression pattern between IR29 and Pok for clusters 7, 10, 14 and 15, whereas most genes had similar expression patterns for clusters 1, 2, 3, 5, 6, 11 and 16. Remerkably, cluster 10 with 142 genes, had higher expression in IR29 compared to Pok, whereas cluster 14 with 62 genes, had higher expression in Pok compared to IR29 (Additional file 2: Figure S1).

### Distinct differences in gene expression profiles of Pok and IR29 without salt stress

**Fig. 3:**
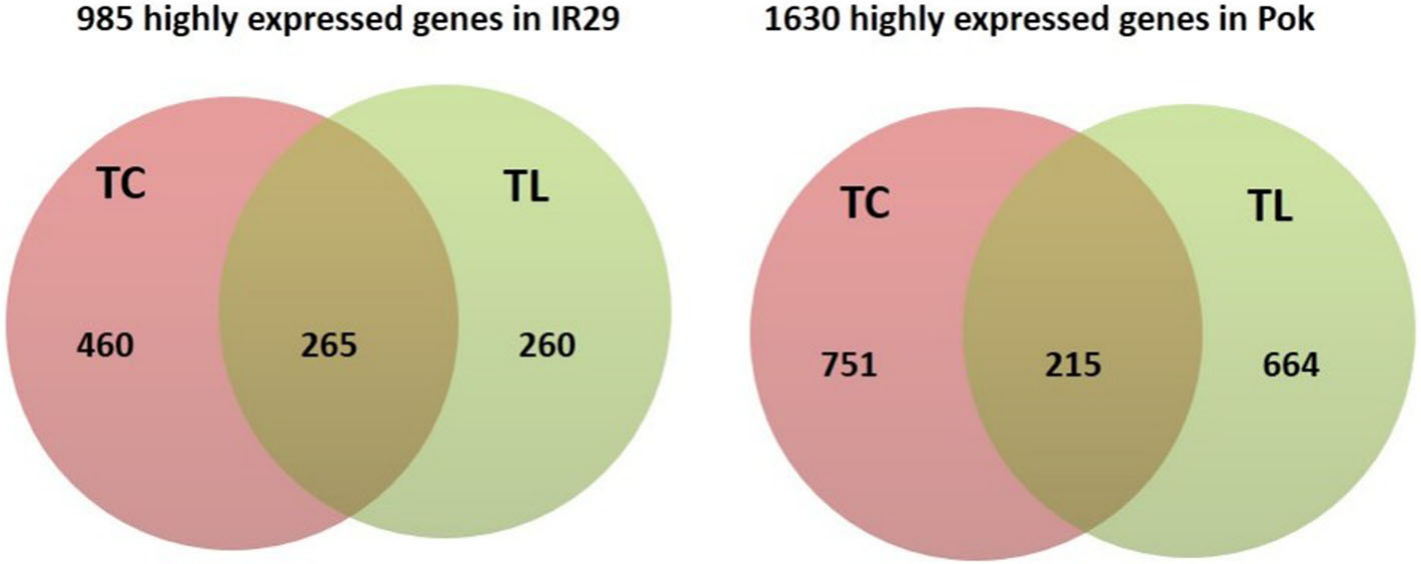
Genes with high basal expression in IR29 and Pok under control conditions. Left: Genes with high expression in IR29 with at least two-fold higher expression and false discovery rate (FDR) < 0.05 as compared with Pok. Right: Genes with high expression in Pok as compared with IR29. TC, transcriptional level; TL, translational level

Differences in gene expression profiles of the transcriptome and translatome without salt stress could offer insights into the contrasting salt tolerance characteristics of Pok and IR29. Therefore, we compared the transcriptome and translatome of plants grown under control conditions. In identifying DEGs we used the following criteria: log_2_ fold change ≥1, at least FPKM ≥5 in one sample for each comparison, and false discovery rate (FDR) ≤ 0.05. This analyses revealed variations between the genotypes (Fig. 3). Without stress, a total of 1630 unique genes showed greater expression in Pok than in IR29 (Fig. 3; Additional file 3: Table S2). Of these, 215 genes were shared between the transcriptome and translatome, whereas 751 and 664 genes were specific to the transcriptome and translatome, respectively. On the other hand, a total of 985 genes showed greater expression under control conditions in IR29 than in Pok; 265 genes were shared between the transcriptome and translatome, and the remaining 460 and 260 genes were specific to transcriptional and translational levels, respectively (Fig. 3 and Additional file 3: Table S2). Indeed, 36.6% of the transcriptionally abundant genes showed a higher abundances at the translational level; however, about half of the highly translated genes did not have a relatively high transcription in IR29. For Pok, 22% of transcriptionally abundant genes showed a higher expression at the translational level but intriguingly, 75.5% of the highly translated genes did not have a relatively high transcriptional regulation. These data revealed a low correlation between transcriptional and translational regulation in both the genotypes under non-stress conditions. More importantly, the transcriptional and translational gene expression profiles were distinct between the two genotypes even without salt stress.

**Fig. 4:**
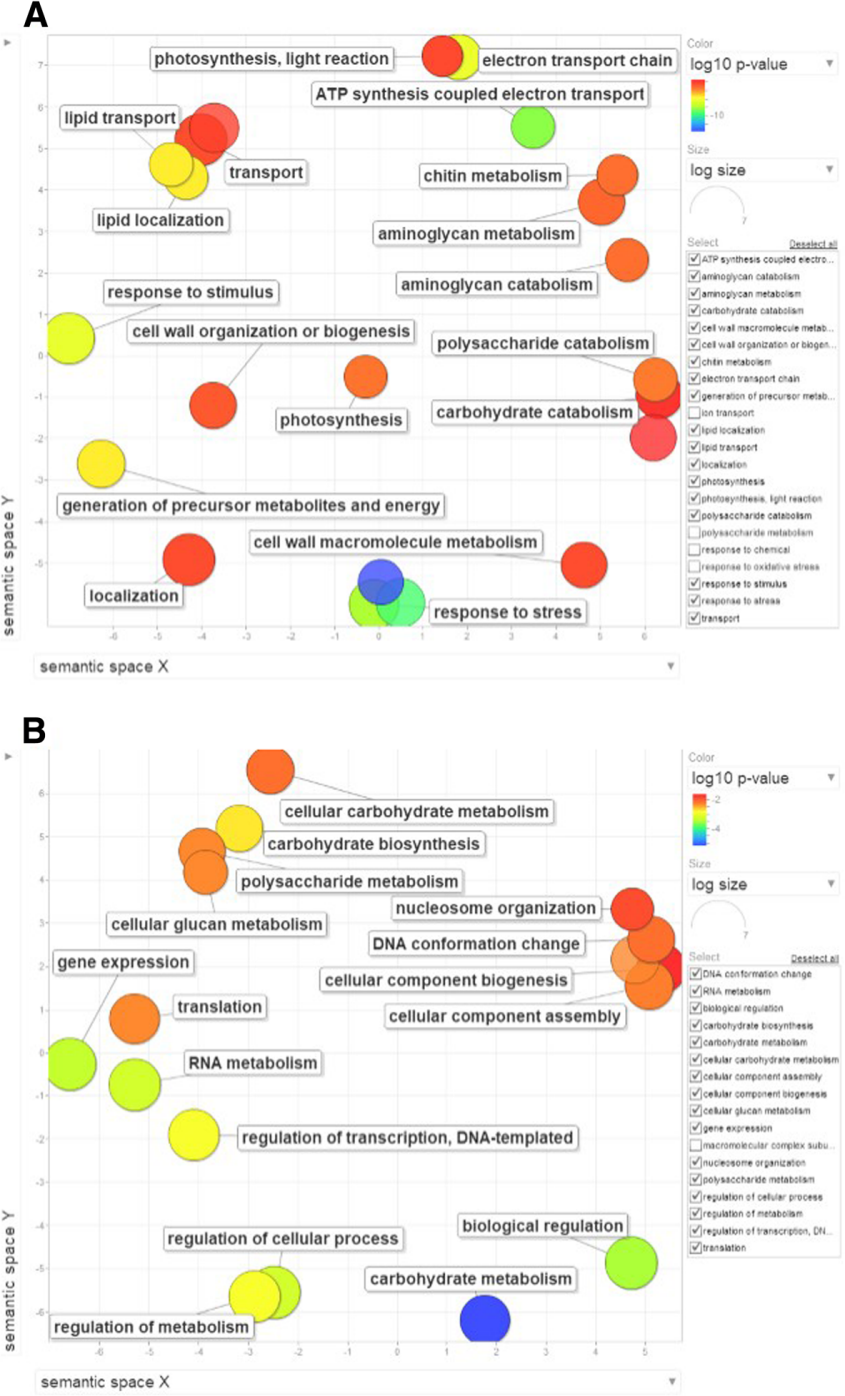
Gene ontology (GO) analysis of genes with high basal expression under control conditions in IR29 (A) and Pok (B) by using REVIGO. The scatterplot shows the cluster representatives (terms remaining after reducing redundancy) in a 2-D space derived by applying multi-dimensional scaling to a matrix of GO term semantic similarities. Bubble color indicates the log10 (*p*-value) for false discovery rates derived from agriGO analysis. The bubble size indicates the frequency of the GO term in the uniprot database (larger bubble size represents more general terms)

To evaluate whether the observed differences in gene expression profiles without stress were biased toward specific functions, we performed GO analysis with agriGO. To remove the redundant GO terms and visualize only the GO differences between the two genotypes, enriched GO categories with FDR < 0.05 were submitted to REVIGO. By using the Uniprot database as a background and the default semantic similarity measurement (SimRel), a clear difference in GO terms was noticed in these genotypes (Fig. 4). Terms overrepresented in Pok include regulation of metabolism, gene transcription, translation, cellular carbohydrate metabolism and DNA conformation. However, terms overrepresented in IR29 were photosynthesis, response to stress, cell wall macromolecule metabolism and organization, transport, and aminoglycan metabolism (Fig. 4). Thus, IR29 and Pok genes differed in certain biological functions when they were not subjected to salt stress. These differences in gene expression profiles between the two genotypes under control conditions might have arisen from the inherent genotypic differences evolved during the domestication process.

### Salt stress-responsive genes in Pok and IR29

**Fig. 5:**
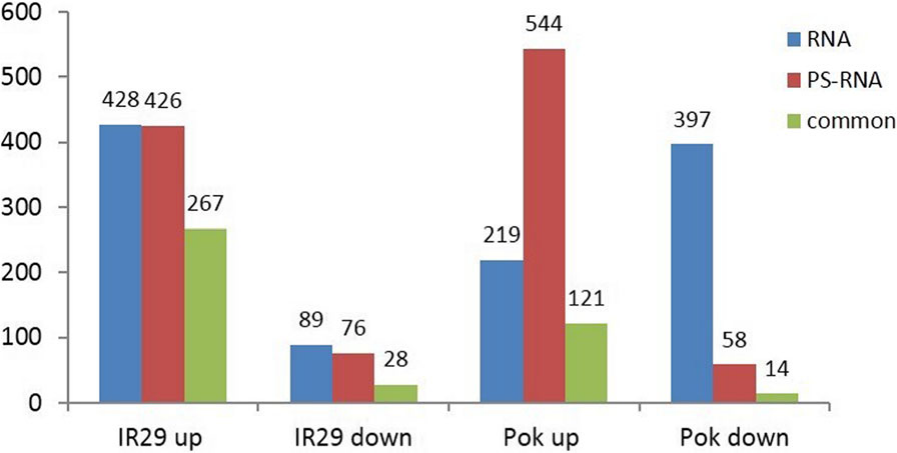
Number of up- and downregulated genes in IR29 and Pok at the transcriptional and translational levels

One of the major objectives of this study was to explore the transcriptional and translational differences when the contrasting salt-tolerant genotypes were exposed to salt stress. To identify salt stress-responsive DEGs at the transcriptional and translational levels in Pok and IR29, we used the same criterion described above (log2 fold change ≥1, at least FPKM ≥5 in one sample of each comparison and FDR ≤ 0.05). This scrutiny has identified 2237 DEGs responding to salt stress in these genotypes (Fig. 5). A complete list of DEGs responding to salt stress in Pok and IR29 at the transcriptional and translational levels is provided (Additional file 4: Table S3).

In IR29, the number of upregulated salt stress-responsive genes was similar at the transcriptional (*n* = 428) and translational (*n* = 426) levels, and more than half of these genes (*n* = 267) were shared between the transcription and translation. Although the number of downregulated genes was much less than that of upregulated genes, the number was approximately similar between transcriptional (*n* = 89) and translational (*n* = 76) levels. Of these, 28 genes were shared between the transcriptome and translatome (Fig. 5). By contrast, in Pok, the number of upregulated genes differed between the transcription and translation, with more upregulated genes at the translational level (*n* = 544) than the transcriptional level (*n* = 219). Among the downregulated genes, fewer genes were responsive at the translational level (*n* = 58) than at the transcriptional level (*n* = 397) (Fig. 5). These observations unambiguously suggest a greater number of upregulated genes were regulated at the translational level in Pok under salt stress. Notably, most of these translationally upregulated genes were unresponsive at the transcriptional level, implying that these genes were more efficiently loaded onto the polysomes for translation. Most of the transcripts in clusters 8 and 15 shown in Additional file 2: Figure S1 belong to this category.

Among the 397 transcriptionally downregulated genes, 14 were common between the transcriptional and translation levels in Pok (Fig. 5). Most of the transcriptionally downregulated transcripts (*n* = 196) did not respond to salt stress at the translational level (log2 fold change vary from − 1 to 1). Interestingly, 26 of these transcriptionally downregulated genes were upregulated at the translational level (log2 fold change > 1); 11 of these including three photosynthesis-related genes (Os01g58038, Os02g24628, Os05g38400), two nicotianamine synthases (Os03g19427 and Os03g19420) and two stress-related genes (Os07g24830, Os05g06500) were significantly upregulated (Additional file 5: Table S4), suggesting that these mRNAs could be stabilized in Pok and/or could be efficiently load onto polysomes for translation despite these were downregulated at the transcriptional level. In fact, differences in polysomal loading between ozone resistant and sensitive *Medicago* genotypes upon ozone stress was reported earlier [24]. Transcripts shown in cluster 7 and some in cluster 16 in Additional file 2: Figure S1 belong to this category. Overall, these results confirmed the previous observation that the changes at the transcriptional level do not always reflect changes in protein level [8–13]. This finding in turn emphasizes that the proteome or translatome analysis especially under stress conditions is important to understand the complete portrait of gene regulation programs.

### Upregulated genes that are shared between transcriptional and translational levels in Pok and IR29

**Fig. 6:**
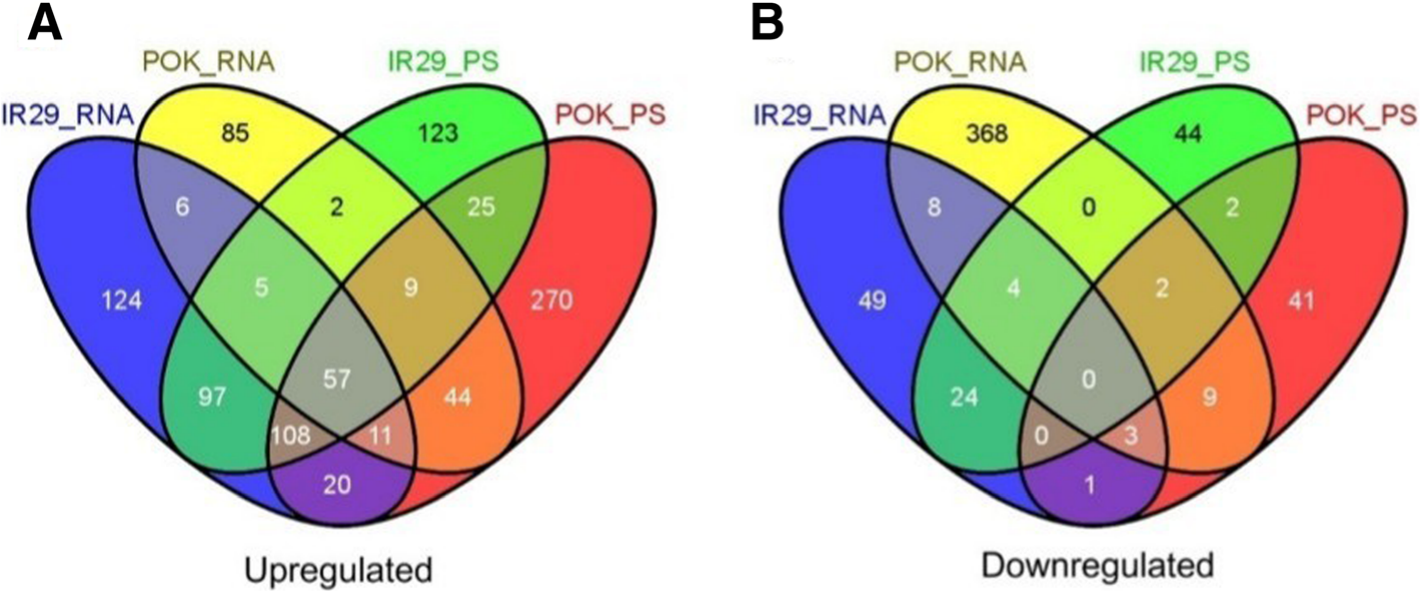
Venn diagram of DEGs at the transcriptional and translational levels between IR29 and Pok genotypes. A: Upregulated genes; b: Downregulated genes

**Fig. 7:**
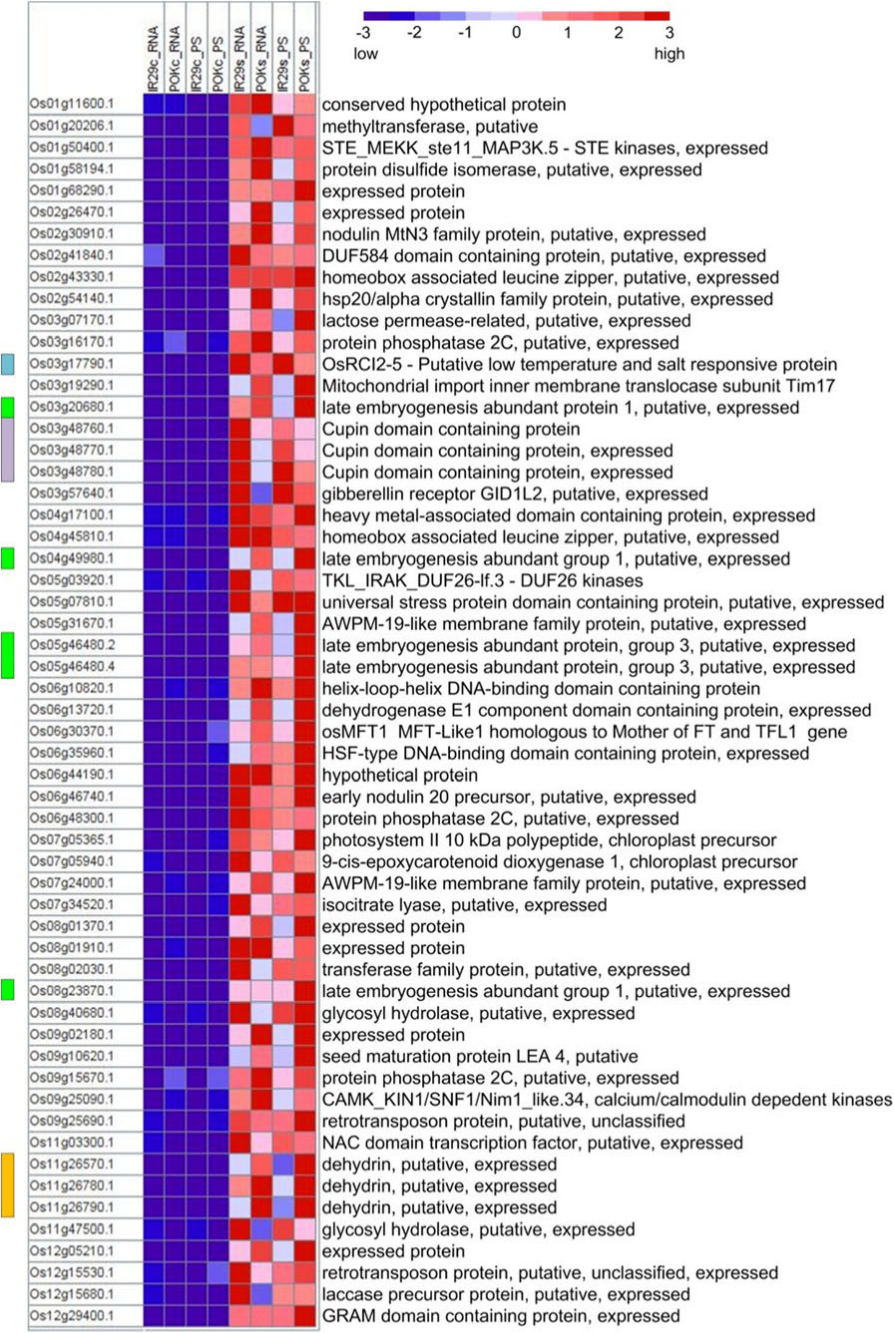
Expression patterns of the 57 upregulated genes that were shared between IR29 and Pok at both the transcriptional and translational levels during salt stress

To identify upregulated genes that were shared between the genotypes under stress, DEGs at the transcriptional and translational levels were compared (Additional file 6: Figure S2). This analysis revealed at least 57 genes that were commonly upregulated both in the transcriptome and translatome of both genotypes in response to salt stress (Fig. 6). Some of these genes belong to the same gene families, and therefore have been grouped into three clusters. The first cluster consisted of three members of the cupin domain-containing protein (CDP) family (Os03g48760, Os03g48770 and Os03g48780), the second cluster included three dehydrin genes (Os11g26570, Os11g26580 and Os11g26590) and the third cluster had five different transcripts of the late embryogenesis abundant protein family (Fig. 7). In addition, a putative low temperature and salt-responsive protein (Os03g17790), a putative universal stress protein domain-containing protein (Os05g07810), a calcium/calmodulin dependent protein kinases (Os09g25090), a NAC domain-containing protein (Os11g03300), two AWPM-19-like membrane family proteins (Os07g24000 and Os05g31670), 9-cis-epoxycarotenoid dioxygenase (Os07g05940) and hsp20/alpha crystallin family protein (Os02g54140.1) were commonly induced at the transcriptional and translational levels in both the genotypes (Additional file 7: Table S5). Most of these genes are well characterized as general stress-responsive genes, and the degree of their induction was often associated with stress tolerance [2]. Consistently, the extent of upregulation of these 57 genes differed between Pok and IR29 (Fig. 7 and Additional file 7: Table S5). The well-known osmotic stress-responsive genes such as the late embryogenesis abundant proteins, dehydrins, AWPM-19-like membrane family protein, hsp20/alpha crystalline family protein, and mitochondrial import inner membrane translocase subunit Tim17 were more strongly upregulated (2- to 7-fold higher) especially at the translational level in Pok compared to IR29. Furthermore, a protein disulfide isomerase (PDI, Os01g58194) and another four unannotated genes were highly upregulated in Pok. Most intramolecular disulfide bond formation in the endoplasmic reticulum is catalyzed by PDI family proteins and functions in multiple biological processes such as oxidative folding of nascent proteins, molecular chaperoning, degradation of abnormal proteins, and redox signaling [25]. The high-level translation of PDI in the Pok genotype may assist in proper protein folding during salt stress.

By contrast, none of the downregulated genes (transcriptionally and translationally) were shared between the genotypes under salt stress (Fig. 6). This finding is similar to a previous study reporting only two downregulated genes shared between IR29 and FL478, a tolerant RIL derived from crossing between Pok and IR29 [21]. The analysis also found 20 DEGs with an opposite regulation pattern between Pok and IR29 upon salt stress (Table 2).

**Table 2:** Top twenty differentially expressed genes (DEGs) that were oppositely regulated by salt stress in IR29 and Pok

locus_id	log_2_FC (IR29s/IR29c)	log_2_FC (IRs-PS/IRc_PS)	log_2_FC (Poks/Pokc)	log_2_FCPoks-PS /Pokc_PS)	gene annotation
Os01g24560.1	down (− 2.44)	no	no	up (2.86)	fruit bromelain precursor
Os02g03900.1	down (−3.45)	no	no	up (1.92)	metal transporter Nramp6
Os01g54910.1	down (−9.57)	no	up (9.24)	no	GTP-binding protein typA/bipA
Os02g40730.1	down (−2.15)	no	no	up (2.98)	ammonium transporter protein
Os10g39980.1	down (−2.43)	no	no	up (1.86)	expressed protein
Os05g02390.1	down (−2.37)	no	no	up (2.81)	ZOS5–02 - C2H2 zinc finger protein
Os11g24140.1	down (−2.20)	no	no	up (2.51)	plastocyanin-like domain containing protein
Os03g19420.2	down (−5.03)	no	down (−4.58)	up (4.25)	nicotianamine synthase
Os03g19427.1	down (−5.62)	no	down (−5.36)	up (3.95)	nicotianamine synthase
Os10g31670.1	down (−2.75)	down (−4.57)	no	up (5.21)	retrotransposon protein, putative, unclassified
Os02g38920.1	no	down (−9.92)	no	up (2.40)	glyceraldehyde-3-phosphate dehydrogenase
Os06g04460.1	no	down (−10.17)	up (10.19)	down (− 10.49)	hypersensitive-induced response protein
Os01g63410.1	no	up (2.52)	no	down (−10.83)	expressed protein
Os06g29120.2	no	up (2.41)	down (−1.99)	no	STE_PAK_Ste20_STLK.5 - STE kinases
Os07g34589.3	no	up (2.58)	down (−12.11)	no	translation initiation factor SUI1
Os08g37790.1	no	up (11.81)	no	down (−11.72)	phospho-2-dehydro-3-deoxyheptonate aldolase
Os01g42650.1	up (11.12)	no	no	down (−12.03)	cytochrome c oxidase subunit 5B
Os02g52860.1	up (2.24)	no	down (−2.21)	no	phosphate carrier protein, mitochondrial precursor
Os03g16950.1	up (2.47)	up (3.38)	down (−1.88)	no	cysteine-rich repeat secretory protein 55 precursor
Os05g43570.1	up (9.77)	up (10.37)	down (−9.57)	down (−9.85)	AGC_AGC_other_NDRh_TRCd.2 - ACG kinases

Number in parenthood means log2(fold change). Up or down means significantly induced or repressed, respectively. Postfix with “-PS” means polysome-associated RNA

### Opposite regulation of salt stress-responsive genes in Pok and IR29

**Fig. 8:**
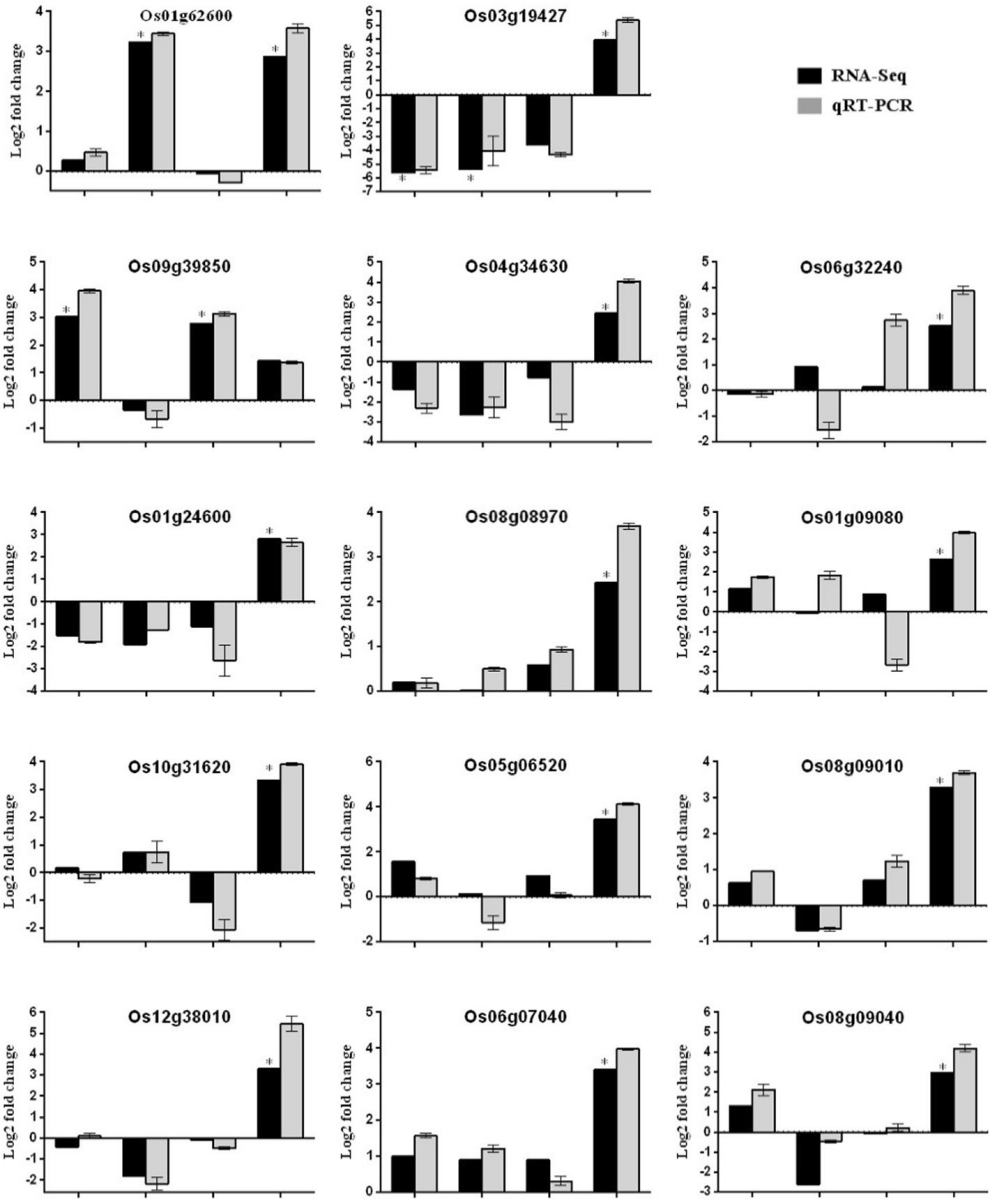
Quantitative RT-PCR validation of DEGs from RNA-Seq data. Y-axis represents log2 fold change or –ΔΔCt value; X-axis represented IR29 and Pok at the total RNA or polysomal (PS) RNA level. Each column represents mean ± SD.* indicates significantly regulated by RNA-Seq analysis

Because Pok and IR29 are distinctly different in their response to salt stress, DEGs that display opposite regulation between them could be expected. The analysis has identified 20 DEGs that displayed an opposite regulation pattern in the two genotypes. Eight genes induced in IR29 at the transcriptional and/or translational level were downregulated in Pok. By contrast, three genes downregulated in IR29 at the translational level were upregulated at the transcriptional or translational level in Pok (Table 2). Similarly, nine of 10 genes downregulated in IR29 at the transcriptional level were upregulated at the translational level in Pok. For instance, two nicotianamine synthase (NAS) genes were repressed at the transcriptional level but upregulated at the translational level in Pok during salt stress. In plants, NAS can catalyze and synthesize niacinamide (NA), which participates in iron ion transportation, distribution and storage, as well as transportation of other heavy metal ions [26]. Overexpression of *OsNAS1* in *Brassica napus* enhanced the protein level of five salt stress-related genes, including dehydrogenase, glutathione S-transferase, peroxidase, 20S proteasome beta subunit, and ribulose-1,5-bisphosphate carboxylase/oxygenase, and significantly improved the plant salt tolerance [26]. On the similar lines, NAS could play important roles in Pok exposed to salt stress. The expression pattern of a NAS gene (Os03g19724) was confirmed by qRT-PCR (Fig. 8).

### Response to salt stress of genes highly expressed under control conditions

Without salt stress, a unique set of genes in Pok and IR29 displayed greater abundance both in the transcriptome and translatome, therefore we examined their response to salt stress. Such genes were represented by 215 and 265 in Pok and IR29, respectively (Fig. 3). Of the 215 high basal expressed genes in Pok, only six were altered at the translational level in Pok; two were upregulated (iron/ascorbate-dependent oxidoreductase-Os06g08041.1, and expressed protein-Os12g13910.1) and four were downregulated (RAN GTPase-activating protein 1-Os05g46560.1, LTPL122-Os04g46830.2, nucleoside-triphosphatases -Os11g25260.1 and Os11g25330.1). Similarly, of the 265 genes with high expression in IR29 under control conditions, 30 were regulated at the translational level in response to salt stress; five genes were upregulated (a proteinase inhibitor II family protein, three SCP-like extracellular proteins and one wound-induced protein) and 25 were downregulated; 17 of these were significantly downregulated at the transcriptional level. Most of these downregulated genes were stress-associated or cell wall-associated genes such as the copper/zinc superoxide dismutase (Os07g46990), peroxidase, glycine-rich cell wall protein, LTP protein and plastocyanin-like protein (Additional file 8: Table S6). Surprisingly, of the 265 high basal expression genes in IR29, 63 were significantly upregulated in Pok at the translational level (Additional file 9: Table S7). A notable example is thionin proteins, which are small cysteine-rich peptides potentially involved in plant pathogen responses [27]. Approximately 10 thionin or thionin-like peptides were highly expressed in IR29 under non-stress conditions and their expression was unaltered or slightly suppressed upon salt stress in IR29. In contrast, these thionin genes were significantly upregulated in Pok at the translational level, which suggests that they may have a role in salt tolerance. Contrastingly, only two genes with high basal expression in Pok were induced by salt stress at the translation level in IR29: one was heat shock protein DnaJ (Os01g42190.1), and the other was an expressed protein (Os04g06520.1).

### **Translational** differences between IR29 and Pok under salt stress

**Fig. 9:**
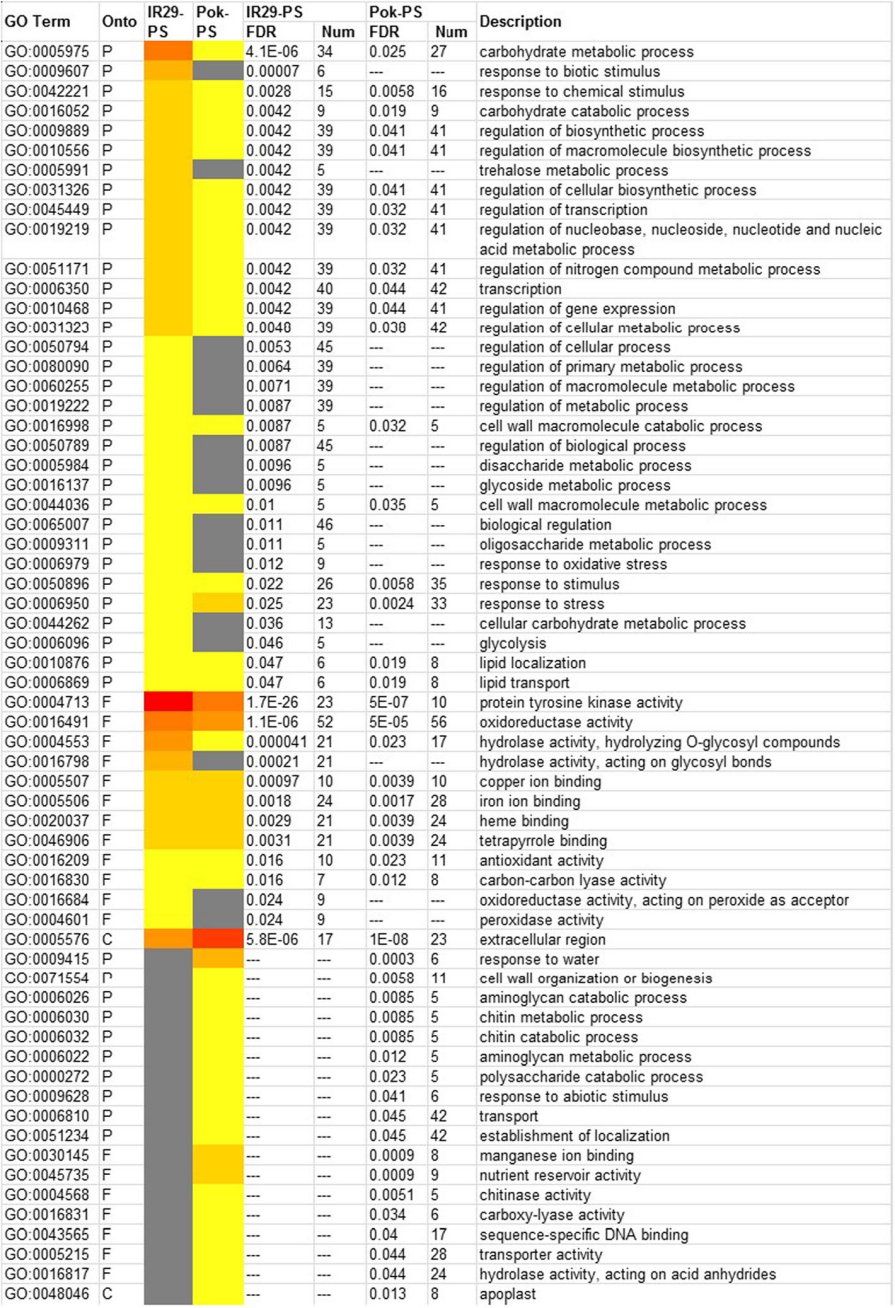
Comparative analysis of enriched GO terms for DEGs in IR29 and Pok at the translational level

**Fig. 10:**
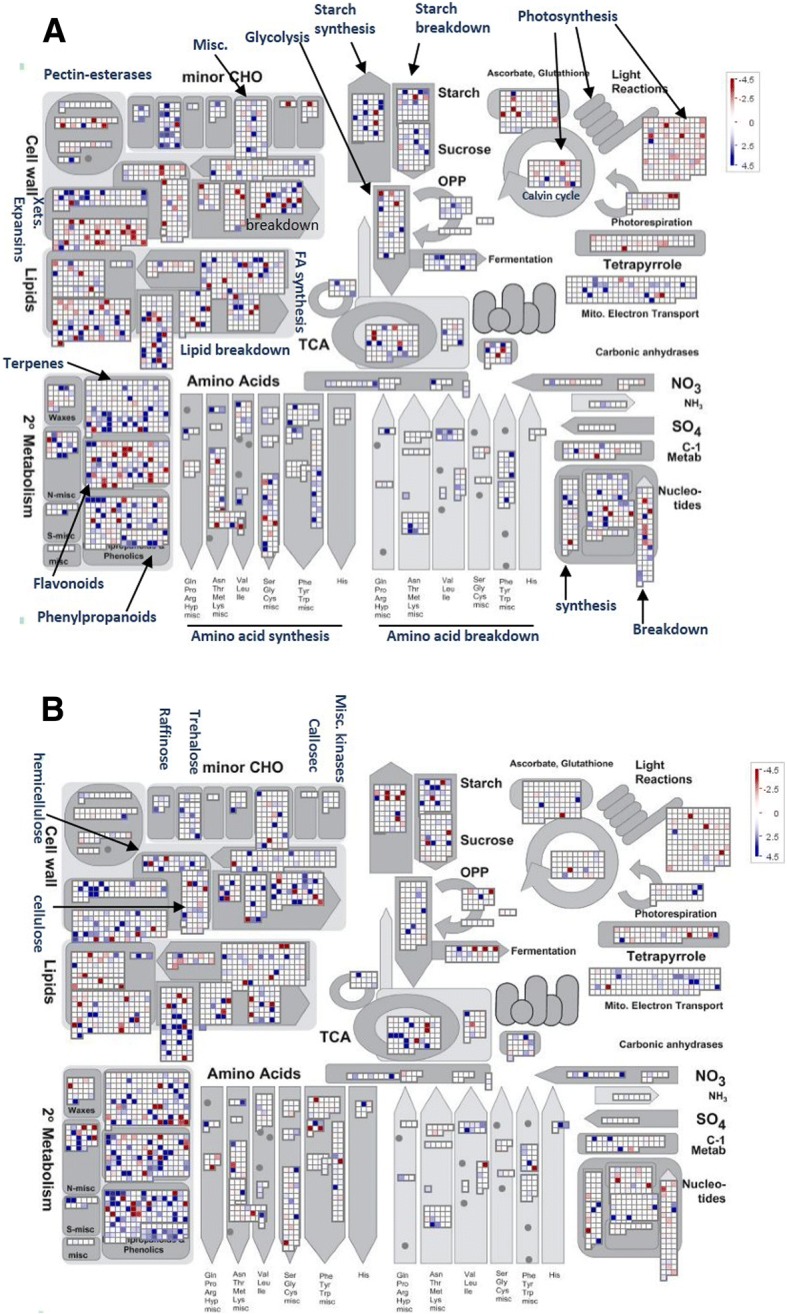
Mapman visualization of salt stress-responsive genes assigned to metabolism at the translational level. A: Salt stress-responsive genes in IR29 at the translational level. B: Salt stress-responsive genes in Pok at the translational level. In panels A and B, the grids represent individual genes. The up- and downregulated genes are indicated in blue and red, respectively. The color brightness represents the degree of difference, as shown in the scale on the right

The mRNAs associated with polysomes represent the translation step of protein synthesis and are therefore a good predictor of protein abundance. To identify translational differences between the genotypes, the DEGs at the translational level were compared. Among the 896 total DEGs, 123, accounting for ~ 13.7% of the total DEGs, were annotated as expressed proteins or hypothetical proteins, similar to a recent report [28]; how these expressed proteins contribute to salt tolerance needs further study. Overall, 203 DEGs were shared between IR29 and Pok, whereas 299 and 399 were specific to IR29 and Pok, respectively (Additional file 6: Figure S2). To assess the differences in the GO enrichment of the translatome in Pok and IR29, batch SEA analysis with agriGO was used. This analysis showed a sharp difference in GO terms between the genotypes (Fig. 9). The GO terms such as the carbohydrate metabolic process, glycolysis, protein tyrosine kinase activity, regulation of biosynthetic process and hydrolase activity were enriched in IR29, whereas response to stress, transporter activity, apoplast, extracellular region and response to water were enriched in Pok (Fig. 9). To further envision the pathways affected by salt stress in IR29 and Pok, we visualized the functional classification of DEGs at the translational level by using MapMan. Overall, the differences between Pok and IR29 were apparent in terms of the pathways related to photosynthesis, cell wall synthesis, and secondary metabolism especially at the translational level (Fig. 10). However, a caution needs to be exercised when extrapolating the gene expression regulation to biological function as suggested by the guilt by association analyses [29], and the identified gene networks and pathways needs further/functional validations.

#### The effect of salt stress on photosynthesis was less severe in Pok

A previous study showed that genes associated with photosynthesis were downregulated within minutes of salt stress but stabilized immediately (within 30 min) in Pok, whereas IR29 almost wilted after 24 h [30]. Consistently, we found the genes associated with photosynthesis were less severely affected in Pok than in IR29 during salt stress (Fig. 10). For example, chlorophyll A-B binding proteins (Os11g13890, Os01g52240.1), ribulose bisphosphate carboxylase small chain (Os12g17600) and plastocyanin-like domain containing proteins (Os07g01440, Os07g35860, Os04g53710) were significantly downregulated in IR29 (Additional file 4: Table S3). Similarly, the genes associated with tetrapyrroles synthesis, light reaction, and Calvin cycle were more repressed in IR29 compared to Pok. FtsH-like chloroplast proteins associated with the attenuation of adverse impacts of Na^+^ on the photosynthetic electron transport chain were accumulated in maize under salt stress [31]. Consistently, this protein (Os02g43350 and Os05g38400) was specifically upregulated in Pok. Another worth pointing gene that is exclusively induced in Pok was transketolase (Os04g19740), which transfers a residue with two carbon atoms from fructose-6-phosphate to glyceraldehydes-3-phosphate, thereby producing ribulose-1,5-bisphosphate (RuBP); this process is important for RuBP regeneration and photosynthesis. The exclusive induction of transketolase at the protein level was consistent with a recent report [28]. Taken together, translationally increased FtsH and transketolase in salt-tolerant Pok likely ensures that the photosynthesis is less affected in Pok under salt stress.

#### Better maintenance of cell wall integrity in Pok under salt stress

Being the first barrier to environmental stress, the cell wall should respond fast by modulating its structure and composition. Therefore, cell wall integrity plays an important role during stress conditions [32, 33]. Different genotypes can share a common mechanism; for example, a similar number of SCP-like extracellular proteins was upregulated by salt stress in Pok and IR29 genotypes (i.e., nine vs ten), which indicates the essential role of SCP-like protein in keeping the cell wall integrity (Additional file 4: Table S3); however, other mechanisms can vary depending on the genotype and its ability to tolerate salinity. Overall, the GO terms cell wall component and organization, and extracellular region were enriched in Pok. The genes associated with cell wall structure were downregulated more in IR29 compared to Pok. The expression of pectin, xyloglucan endotransglucosylase/hydrolase, cellulose and hemicellulose was severely repressed by salt stress in IR29 at the translational level, whereas translation of these genes in Pok was less affected (Fig. 10). Other cell wall-related genes such as the expansin precursor, hydroxyl proline-rich glycoprotein, glycine rich cell wall structural protein, and CDP were exclusively induced in Pok.

Expansins are cell wall proteins that provide the required strength for plant growth and development. Recently, it was shown that the expansins could improve plant stress tolerance [34]. Furthermore, the expansins tend to increase their abundance in salt-tolerant compared to salt-sensitive maize [35]. Consistent with these observations, three expansins (Os01g14650, Os02g16730, Os05g19570) were highly upregulated in Pok under salt stress, whereas at least one expansin (Os06g01920) was repressed in IR29. The gene encoding arabinogalactan protein (Os06g21410) was upregulated in Pok, which is consistent with a previous report [21]. Three glycine-rich proteins (Os04g56040, Os10g31620, Os01g06310) were upregulated in the translatome of Pok, whereas three other related glycine-rich cell proteins (Os10g31530, Os10g31710, Os10g31660) were repressed in IR29. Cupin domain-containing protein (CDP) possesses biochemical activities associated with the cell wall. The induction of CDP3.1 and CDP3.2 was suggested to improve seed germination and seedling growth under salt stress [36], and consistent with this, more CDP proteins (eight in Pok but only four in IR29) were upregulated in the translatome of Pok as compared with IR29 (Additional file 4: Table S3); the upregulation of three CDPs was confirmed by qRT-PCR (Fig. 8). The basal expression of peroxidase precursors (Os05g04490.1, Os05g06970.1, Os04g34630.1 and Os10g02070.1) was higher in IR29 compared to Pok; however, their expression was unaltered by salt stress in IR29 but was upregulated in Pok at the translational level. The peroxidase is critical for lignin biosynthesis, and induced expression of peroxidase could contribute to increased lignin accumulation, which could promote cell wall rigidity during salt stress. Overall, these results suggest that Pok can maintain better cell wall plasticity than IR29 under salt stress.

#### Transporter genes responding to salt stress

We found many transporter genes responding to salt stress in Pok and IR29; however, the GO term transport (GO:0006810) was enriched only in Pok. A metal transporter gene (Os02g03900) was exclusively upregulated in Pok, which infers a role in salt tolerance, as reported earlier [37]. Pok showed upregulation of amino acid permease protein (Os04g35540), ammonium transporter protein (Os02g40730), heavy metal-associated domain-containing proteins (Os01g61070, Os01g74490), trafficking protein particle complex subunit (Os06g04900, Os07g27150), two major facilitator superfamily antiporters (Os01g16260, Os11g04104), and mitochondrial import inner membrane translocase subunit Tim17 (Os01g19770) (Additional file 4: Table S3). Aquaporins are known as pore-forming membrane proteins that belong to a large family of major intrinsic proteins (MIPs) that transport water and low-molecular-weight neutral compounds across the membrane. Rice has 33 aquaporin genes that include 11 plasma-membrane intrinsic proteins (PIPs) [38]. In rice, salt stress-induced regulation of aquaporins may be associated with salt tolerance [39]. The overexpression of PIP aquaporins can confer tolerance to various abiotic stresses in rice [40, 41]. In our study, IR29 showed downregulation of an aquaporin gene *(Os02g51110)*, but Pok showed upregulation of two aquaporins *(Os02g41860 and Os01g8660)* in the translatome (Additional file 4: Table S3). These results indicate that Pok is more active than the IR29 genotype in translocating molecules across the membranes.

#### Increased antioxidant systems in Pok under salt stress

Like most abiotic stresses, salt stress induces oxidative stress by increasing ROS accumulation, which negatively affects cellular processes [2]. ROS in the form of O_2_^_^, H_2_O_2_, and *OH can damage almost all macromolecules of the cell and can lead to cell death. To decrease the intensity of oxidative stress, plant cells increase both enzymatic (catalase, superoxide dismutase, ascorbate peroxidase, glutathione reductase, and glutathione-S-transferase) and non-enzymatic antioxidant (alpha-tocopherol, B-carotene, glutathione, and ascorbate) systems [42]. The downregulation of copper/zinc superoxide dismutase (Os07g46990) and peroxidases observed in IR29 at the translational level indicates that IR29 has decreased ability to reduce oxidative stress (Additional file 8: Table S6), which is consistent with the finding in IR64, another salt-sensitive rice genotype [28]. In the present study, four glutathione S-transferase (GST) genes (Os06g12290, Os10g38340, Os01g49720, Os10g38360) were commonly upregulated in both IR29 and Pok at the translational level. This finding is consistent with a previous study reporting similar responses in rice exposed to salt stress [43]. Additionally, six GSTs (Os01g25100, Os01g49710, Os01g72160, Os01g72150, Os01g72170 and Os10g38350) were exclusively upregulated in Pok (Additional file 4: Table S3). Plant GSTs are known to detoxify xenobiotics and hydroperoxides and function in abiotic stress responses [44]. Overexpression of rice GST genes in Arabidopsis confers tolerance to salt and other abiotic stresses [45, 46]. Upregulation of a greater number of GST genes at the translational level could aid Pok in decreasing oxidative stress levels. Furthermore, metallothioneins (Os03g17870 and Os12g38010), also known for their ROS scavenging activity, were specifically upregulated in the translatome of Pok. Indeed, overexpression of the metallothionein OsMT1-eP has been shown to enhance tolerance to abiotic stresses including salinity [47]. Another key enzyme specifically induced in Pok at the translational level was iron/ascorbate-dependent oxidoreductase (Os06g07941, Os06g08041). Taken together, the upregulation of major antioxidant genes in Pok at the translational level suggests its robust antioxidative capacity during salt stress, whereas such responses were lacking or minimal in IR29.

#### Transcription factors responding to salt stress

The transcription factors (TFs) play a crucial role in regulating the expression of stress-responsive genes. The expression of several TFs such as the MYB, bHLH, NAC, ERF, WRKY and bZIP factors is altered under salt stress [48]. In our study, 59 TFs were identified as DEGs responding to salt stress in IR29 and Pok at the translational level. TFs such as MYB, WRKY, AP2 and NAC were represented by the highest numbers (Table 3). Approximately 24 TFs, including seven MYBs, three WRKYs, three homeobox associated leucine zippers, two AP2 genes, four NAMs and two NACs, were commonly regulated in both IR29 and Pok. Meanwhile, 16 and 19 TFs were uniquely regulated in IR29 and Pok, respectively (Table 3). Two ethylene-responsive TFs, an Auxin response factor, and one heat-stress TF were uniquely regulated in IR29 (Additional file 10: Table S8); one bZIP, one Auxin-responsive Aux/IAA gene family member, and three HBP-1b (leucine-zipper type) TFs were specifically regulated under salt stress in Pok (Additional file 10: Table S8). The regulation pattern of AP2 (Os09g39850), WRKY (Os01g09080), Auxin-responsive factor (Os06g07040) and bHLH (Os05g06520) were confirmed by qRT-PCR (Fig. 8). The upregulation of these TFs may be significant in regulating the downstream salt stress-responsive genes [48–51].

**Table 3:** Transcription factors responding to salt stress in IR29 and Pok at the translational level

Transcription factor family	Shared	IR29-specific	Pok- specific	Total number
AP2 domain containing proteins	2	4	2	8
Auxin response factors	0	1	0	1
Basic helix-loop-helix proteins	2	0	2	4
bZIP transcription factors	0	0	1	1
Ethylene-responsive transcription factors	0	2	0	2
Heat stress transcription factors	0	1	0	1
HSF-type DNA-binding domain containing proteins	1	0	3	4
Homeobox associated leucine zipper	3	0	1	4
MYB family transcription factor	7	2	0	9
NAC domain transcription factor	2	1	0	3
No apical meristem protein	4	2	3	9
Aux/IAA gene family members	0	0	1	1
WRKY transcription factors	3	3	3	9
Transcription factor HBP-1b	0	0	3	3
Total	24	16	19	59

#### Other stress-responsive genes

Abscisic stress-ripening (ASR) was reported to be upregulated in response to abiotic stress; in the present study, two ASRs (Os01g72910.1 and Os01g72900.1) were exclusively upregulated in Pok at the translation level, which is consistent with previous reports [30, 52].

### Salt stress-responsive genes localizing to the known salt-tolerant quantitative trait loci (QTL)

Salt tolerance is a quantitative trait, and mapping studies using Pok and IR29 inbred populations identified SalTol as a major QTL that regulates Na-K balance in shoots of seedlings [53]. To gauge the link between DEGs of the translatome and salt tolerance, we mapped these genes to the known QTL of salt tolerance (www.gramene.org). In total, 36 and 43 DEGs could be localized to the 11 salt-related QTL for IR29 and Pok, respectively; 18 were shared between the genotypes, whereas 18 and 25 were specific to IR29 and Pok, respectively (Additional file 11: Table S9)*.* In this *Saltol* QTL region, methyltransferase (Os01g20206), pectinesterase (Os01g20980) and glutathione S-transferase (Os01g25100) were among the commonly upregulated genes in IR29 and Pok; two jacalin-like lectin domain-containing proteins (Os01g24710, Os01g25280) were exclusively induced in IR29 but not in Pok (Additional files 11 and 12: Tables S9 and S10). These results are similar to previous results [21]. OsHKT1 (Os01g20160) functions as a low-affinity Na^+^ transporter especially under saline conditions and is a major candidate gene underlying major QTL (SalTol) [19]. Cotsaftis et al. [20] reported that *OsHKT1* was highly induced by salt stress in roots of IR29 compared to Pok, whereas Walia et al. [22] showed higher induction of *OsHKT1* in FL478, a salt-tolerant RIL derived from a IR29 and Pok cross. In our study, *OsHKT1* expression was almost unaltered in response to salt stress in both genotypes at the transcriptional level. *OsHKT1* expression was slightly repressed in IR29 by salt stress, but was highly upregulated at the translational level in Pok. The discrepancies between different studies may result from differences in tissue sources (whole seedlings were used in this study whereas Walia et al. [22] used shoots and Cotsaftis et al. [20] analyzed root tissue), development stages, growth conditions, severity and duration of salt stress.

The DEGs of the translatome were also mapped to QTL derived from Pok and IR29 mapping populations [54, 55]. In total, 83 and 87 DEGs were found for IR29 and Pok, respectively; 39 were shared between IR29 and Pok (Additional file 12: Table S10). The QTL on chromosome 1 (Qsnc1, Qskc, qsnk1, qrkc1 and qrnk1) control root and shoot Na^+^/K^+^ ratio. An ATPase (Os01g19260) and mitochondrial import inner membrane translocase subunit Tim17 (Os01g19770) coding genes were mapped on these QTL and were exclusively upregulated in Pok. Another QTL (qrnk9, qsnk9 and qses9) controls root and shoot Na^+^/K^+^ ratio. Two F-box domain-containing proteins (OsFBX319 and OsFBX322) exclusively mapped onto this QTL were upregulated in Pok during salt stress (Additional file 12: Table S10). These observations interconnect the genetic mapping results with the RNA-Seq results of the present study and further support the salt tolerance trait of Pok.

### Validation of RNA-Seq analysis using quantitative RT-PCR

To validate the expression profiles obtained by RNA-Seq, we used quantitative RT-PCR (qRT-PCR). The regulation pattern of a randomly selected 19 DEGs was validated. In most cases, the results were consistent between the RNA-Seq and qRT-PCR, which indicates that the RNA-Seq data were largely reliable and truly reflected the transcription and translation profiles. In general, upregulated genes were more consistent and comparable to RNA-Seq data than were downregulated genes (Fig. 8).

### Identification of novel transcripts and alternative splice variants

RNA-Seq can identify novel transcripts that are transcribed in the genome. In both RNA-Seq (transcriptome) and polysomal RNA-Seq (translatome) libraries, thoudands novel regions that were transcribed were scored. The sequence and blast information of these novel transcripts are shown in Additional file 13: Table S11. In the IR29 transcriptome, a total of 1250 novel loci/genes were detected. Using the same criteria described for identifying DEGs, 35 salt-responsive novel transcripts were found. In the Pok transcriptome, 838 novel transcripts were detected; 38 were responsive to salt stress. In the translatome of IR29, 878 novel transcripts were detected; 31 were responsive to salt stress. Similarly, in the Pok translatome, 539 novel transcripts were detected; only nine responded to salt stress (Additional file 1: Table S1).

Alternative splicing is another important gene regulatory mechanism associated with plant stress responses [56]. The TFs and other genes associated with stress tolerance were reported to undergo alternative splicing in Arabidopsis and other plants [20, 26, 57, 58]. In rice, salt stress has been reported to change the expression of alternatively spliced *AOX* [20, 59]. Similarly, several TFs such as *OsDREB2B* are alternatively spliced and undergo expressional changes under salt, temperature and drought stress [60]. In the present study, more than 7000 alternate splicing events were detected within the assembled transcripts of IR29 and Pok. At least eight alternatively spliced transcripts (two beta-expansin precursor isoforms, a dehydrin, a wound-induced protein precursor, and a photosynthesis related photosystem II polypeptide) were regulated by salt stress (Additional file 14: Table S12). Dehydrin is an important stress-associated gene family, and a dehydrin isoform (Additional file 15: Figure S3A) (TCONS_00081900, Os11g26790.1) was upregulated in IR29 and Pok at both transcriptional and translational levels. Interestingly, the induction level was comparable between IR29 and Pok at the transcriptional level but was 13-fold greater at the translational level in Pok compared to IR29. Beta-expansins are important in mediating growth, and their abundance has been found to differ between salt-tolerant and salt-sensitive genotypes of maize [61]. In the present study, two isoforms of beta-expansin precursor (Os09g29710) were upregulated at the transcriptional and translational levels in response to salt stress in IR29, however, no significant induction was detected in Pok (Additional file 14: Table S12). A splice variant of wound-induced protein precursor (TCONS_00079570, Os11g37970.1) was upregulated at the transcriptional level but not at the translational level in both IR29 and Pok. Similarly, a splice variant of photosystem II 10 kDa polypeptide (Os07g05360.1) (Additional file 15: Figure S3B) showed differential abundance in only Pok in response to salt stress (Additional file 14: Table S12). A list of alternatively spliced genes, including structural genes (β-expansins) and stress-associated genes identified in the transcriptome and translatome of Pok and IR29 adds to the alternatively spliced gene database of rice and is also an important resource for investigating their roles in salt tolerance.

## Conclusions

Salt stress is a complex process involving many genes related to biological, molecular and cellular pathways. The identification of genes that are regulated in response to salt stress would provide a better understanding of the molecular processes and useful for improving the salt tolerance of rice via molecular breeding and/or transgenic strategies. Although many salt stress-responsive genes have been identified by transcriptome analysis in Pok and IR29, however, whether all of these transcriptionally regulated genes translate into proteins or not is unkown in these contrasting rice genotypes. The proteins are direct effectors of plant stress responses. Proteins include enzymes and also components of transcription and translation; they regulate plant stress responses at both mRNA and protein levels [62]. Previous studies have demonstrated that changes at the transcription level do not often correspond to changes at the protein level [63, 64]. Polyribosomal loading of transcripts is the most important step in the translation initiation process. By examining transcriptome and translatome, the qualitative and quantitative differences in the transcriptional and translational profiles of Pok and IR29 even without salt stress was noted in this study. Furthermore, upon salt stress, translational regulation sharply differed between the Pok and IR29; Pok showed the highest number of upregulated genes, and a far less number of downregulated genes than that for IR29 at the translational level. These findings suggest a potential yet unknown mechanism contributing to the stability and efficient loading of the mRNAs in Pok; this distinct regulation was less evident in IR29.

The present study also has identified a set of shared genes that are responding to salt stress regardless of genotype and also identified a number of genes that are not known to differ in their regulation during stress treatment. For instance, protein disulfide isomerase, NAS genes, thionins, and several uncharacterized proteins could be added to the category of stress responsive genes in plants. This study also identified many novel transcripts and alternative splice variants that respond to salt stress.

Overall, sequencing of polysome-associated RNA offered major differences in gene regulation operating at the translational level between IR29 and Pok. These findings suggest that the salt tolerance of Pok could be attributed to its upregulation of genes, especially at the translational level, associated with cell wall synthesis, ROS scavenging, translocating molecules, and TFs, and better maintenance of photosynthesis-related genes.

## Methods

### Plant materials and salt stress treatment

Rice genotypes IR29 and Pok seeds were germinated between moist paper towels and the germinated seedlings were transferred to 96-well PCR plates with the bottom cut off and placed to float on Yoshida medium [65]. Rice seedlings were grown in a controlled growth chamber (22–24 °C) with a 16-h/8-h (day/night) photoperiod and 300 μmol m^− 2^ s^− 1^ light intensity. Approximately 24-day-old seedlings were subjected to salt stress with supplementation of 150 mM NaCl for 24 h, and control samples remained on Yoshida medium. Whole seedlings of control and salt stress-treated samples were harvested and used for isolation of polysomes as well as the total RNA.

### Isolation of the polysomal fractions

The polysomal fractions from the Pok and IR29 samples were isolated as described [66]. Briefly, rice seedlings were ground into a fine powder with liquid nitrogen. Approximately 500 μL powder was transferred to a 2-ml tube containing 1250 μL polysome extraction buffer (200 mm Tris (pH 9.0), 200 mm KCl, 26 mm MgCl_2_, 25 mm EGTA, 100 μm 2-mercaptoethanol, 50 μg ml^− 1^cycloheximide, 50 μg ml^− 1^ chloramphenicol, 1% (*v*/v) Triton X-100, 1% (v/v) Brij-35, 1% (v/v) Tween-40, 1% (v/v) non-iodent P-40, 2% (v/v) polyoxyethylene 10 tridecyl ether, 1% (v/v) deoxycholic acid) and thoroughly mixed by using a spatula and placed on ice for 10 min with occasional mixing by inverting tubes. The mixture was spun for 2 min at 14000 rpm (4 °C) and the supernatant was passed through a QIA shredder (QIAGEN) column by centrifugation for 1 min at 14000 rpm (4 °C). Approximately 600 μL of the sample was layered on top of the sucrose density gradients (20–60% sucrose, *w*/*v*) and centrifuged for 120 min at 40,000 rpm (275,000×g) in a Beckman OPTIMA LE-80 centrifuge. The optical density (OD) of the samples was measured by using a UA-5 detector and a Gradient Fractionator (model 640, ISCO) reading absorbance throughout the sucrose gradient at 254 nm. Fractions with more than two ribosomes were pooled and used for RNA isolation with Trizol reagent.

### Total RNA isolation and poly (a) RNA purification

Total RNA from the Pok and IR29 samples was isolated by using Trizol Reagent according to the manufacturer’s instructions (Invitrogen). RNA quality was tested by using BioAnalyzer (Agilent). Equal quantities of RNA from three biological replicates were pooled for each treatment. Poly (A) RNA was isolated from 10 μg each of total RNA and polysomal RNA by using the MicroPoly (A) Purist kit (Ambion).

### mRNA-Seq library construction and sequencing

mRNA-Seq libraries were generated by following the Illumina mRNA Sequencing Sample Preparation Guide. Briefly, poly (A) RNA was fragmented into small pieces by using divalent cations at 94 °C for 5 min. The fragmented mRNA was converted into cDNA by using random-primers and reverse transcriptase; after 2nd-strand cDNA synthesis, end repair and A-tailing, pair-end adaptors were ligated to cDNA ends. Ligated DNA was separated on a 2% agarose gel and DNA in the 200-bp (±25 bp) range was excised, gel-purified and enriched by PCR amplification with 15 cycles. After PCR product purification and quality testing by using BioAnalyzer (Agilent), mRNA-Seq libraries were sequenced by Illumina HiSeq 2000. In total, eight mRNA-Seq libraries were generated and sequenced (IR29c, IR29c-ps, IR29s, IR29s-ps, POKc, POKc-ps, POKs and POKs-ps, representing samples of IR29 control, polysomal fractions of IR29 control, IR29 salt stress, polysomal fractions of IR29 salt stress, Pok control, polysomal fractions of Pok control, Pok salt stress and polysomal fractions of Pok salt stress, respectively).

### Computational analysis of sequencing libraries

A self-written program was used to choose reads with ≤5 low-scored nucleotides (< 10) and simultaneously with ≤5 unspecified nucleotides (marked as “N” in the raw data) among the 50- to 51-nt sequencing reads, which were discarded. The remaining reads were analyzed with Cufflinks [67]. Briefly, TopHat [68] was used to align the reads to the genome of rice (MSU v6.1) with option “-G”. Then, Cufflinks was used to assemble the alignments from TopHat, and assembled transcripts from the individual libraries were merged by using Cuffmerge. Cuffcompare was then used to compare the combined transcripts to annotated transcripts in the MSU Rice Genome Annotation Database (v6.1). Finally, Cuffdiff was used to compare the transcript levels in the control and salt stress libraries. The transcripts simultaneously satisfying the following criteria were defined as differently expressed genes (DEGs): (1) sufficient mapped reads (marked as “OK” by Cuffdiff); (2) absolute value of log2 fold change ≥1; (3) FDR-corrected *P* -value < 0.05; and (4) at least 5 FPKM in one condition of each comparison. The cDNA, exon, intron and intergenic sequences were downloaded from the MSU Rice Genome Database (v6.1). The filtered reads were also aligned to different categories of molecules with SOAP2 [69] by allowing at most 2 mismatches. The alignment results of SOAP2 were then used to calculate the number of reads mapped to cDNAs, exons, introns and exon-intron junctions.

### GO enrichment analysis

DEGs were used for Gene Ontology enrichment analysis with agriGO v2.0 (http://systemsbiology.cau.edu.cn/agriGOv2/) [70] and REduce and Visualize GO (REVIGO) (http://revigo.irb.hr/) [71]. To thoroughly visualize the metabolic pathways responding to salt stress at the translational level in IR29 and Pok, we further mapped the DEGs obtained in the translatome to terms in the MapMan database and inspected for significantly enriched pathway terms compared to the genome background [72].

### Validating the gene expression profiles by using quantitative RT-PCR

Quantitative RT-PCR was performed to validate DEGs in the same RNA samples used for the RNA-Seq analysis. The isolated total RNA was treated with DNase I and reverse transcribed by using an oligo-dT primer and Superscript II reverse transcriptase (Invitrogen) according to the manufacturer’s instructions. Primers were designed to amplify ~ 150-bp fragments. Quantitative PCR was carried out using Maxima SYBR Green qPCR Master Mix (Thermo Scientific) and 7500 Real-Time PCR System (Applied Biosystems). All samples were run in triplicate and each cDNA sample was run in duplicate; actin was used as the reference gene, and the mean -∆∆Ct values were plotted with standard deviation. Primers used in this study are listed in Additional file 16: Table S13.

## Additional files

Additional file 1: **Table S1**. Distribution of assembled transcripts to different categories. (XLSX 11 kb)

Additional file 2: **Figure S1.** Overview expression patterns of transcriptome and translatome of IR29 and Pok under control and salt stress conditions. The purple line represents the mean expression level of the genes in the same cluster; grey lines represent the expression of each gene in the cluster. Transcripts were divided into 16 clusters (cluster 1 to 16) based on their expression level by using k-Means Clustering (KMC). The vertical axis represents the gene expression value (log2 FPKM), and the horizontal axis represents the 8 samples (in order from left to right: IR29c_RNA, IR29s_RNA, Pokc_RNA, Poks_RNA, IR29c_PS, IR29s_PS, Pokc_PS and Poks_PS).Most genes in clusters 1, 2, 3, 6 and 16 had similar expression in IR29 and Pok but were not responsive by salt stress. Cluster 5 represents genes that were moderately upregulated by salt stress, cluster 9 represents genes that were strongly upregulated by salt stress in both IR29 and Pok at the transcriptional and translational levels, and cluster 13 represents genes were repressed by salt stress in IR29 and Pok at both transcriptional and translational levels. Cluster 10 represents genes with higher abundances at both transcriptional and translational levels in IR29 but extremely low abundance in Pok, whereas cluster 14 represents genes with very low expression at both transcriptional and translational levels in IR29 under both control and salt stress conditions but higher transcriptional and translational levels in Pok; these genes did not respond to salt stress. Cluster 7 represents genes that did not respond to salt stress at the transcriptional level in Pok but were highly upregulated at the translational level. Clusters 8 and 15 represent genes highly upregulated by salt stress in IR29 at the transcriptional and translational levels but only upregulated in Pok at the translational level. Cluster 11 represents genes with relatively higher expression in Pok than in IR29 at the transcriptional level. Cluster 12 represents genes with lower translation than transcription. (JPG 129 kb)

Additional file 3: **Table S2.** Highly expressed genes of Pok and IR29 under control conditions. (XLSX 17980 kb)

Additional file 4: **Table S3.** Expression of salt stresss responsive genes in IR29 and Pok at transcriptional and translational levels. (XLSX 277 kb)

Additional file 5: **Table S4.** Genes downregulated at the transcriptional level but upregulated at the translational level in Pok. (XLSX 14 kb)

Additional file 6: **Figure S2.** Comparative analysis of DEGs between IR29 and Pok at the transcriptional level (A) and translational level B). (JPG 65 kb)

Additional file 7: **Table S5**. 57 commonly upregulated genes in Pok and IR29 both at the transcriptional and translational levels. (XLSX 22 kb)

Additional file 8: **Table S6**. The salt stress-responsive translational regulation of the highly expressed genes at basal level in IR29. (XLSX 16 kb)

Additional file 9: **Table S7.** The salt stress-responsive translational regulation of genes in Pok which were highly expressed at basal level in IR29. (XLSX 21 kb)

Additional file 10: **Table S8**. Transcription factors differentially regulated by salt stress in IR29 and Pok at the translational level. (XLSX 27 kb)

Additional file 11: **Table S9**. DEGs within the salt tolerant quantitative trait loci (QTL) (www.gramene.org). (XLSX 636 kb)

Additional file 12: **Table S10**. DEGs within QTL identified from IR29/Pok population. (XLSX 20 kb)

Additional file 13: **Table S11**. Details of novel intergenic transcripts. (XLSX 635 kb)

Additional file 14: **Table S12**. Significantly regulated alternative splice variants in IR29 and Pok. (XLSX 15 kb)

Additional file 15: **Figure S3.** Novel alternate splice variants of dehydrin (Os11g26790) (A) and photosystem II 10-kDa polypeptide, chloroplast precursor (Os07g05360) (B). (JPG 112 kb)

Additional file 16: **Table S13.** Primer sequences used for qPCR validation. (XLSX 10 kb)

## Abbreviations

DEG: differentially expressed genes
FDR: false discovery rate
FPKM: fragments per kilobase of transcripts per million mapped reads
GO: gene ontology
Pok: Pokkali
QTL: quantitative trait loci
ROS: reactive oxygen species
TFs: transcription factors

## References

[1] Machado R, Serralheiro R (2017). Soil salinity: effect on vegetable crop growth. Management practices to prevent and mitigate soil salinization. Horticulturae.

[2] Bartels D, Sunkar R (2005). Drought and salt tolerance in plants. Crit Rev Plant Sci.

[3] Zhu J-K (2002). Salt and drought stress signal transduction in plants. Annu Rev Plant Biol.

[4] Hasegawa PM, Bressan RA, Zhu J-K, Bohnert HJ (2000). Plant cellular and molecular responses to high salinity. Annu Rev Plant Physiol Plant Mol Biol.

[5] Siddiq EA, Vemireddy LR, Nagaraju J (2012). Basmati Rices: genetics, breeding and trade. Agricultural Research.

[6] De Leon TB, Linscombe S, Subudhi PK (2017). Identification and validation of QTLs for seedling salinity tolerance in introgression lines of a salt tolerant rice landrace ‘Pokkali’. PLoS One.

[7] Mandal AB, Chowdhury B, Sheeja TE (2000). Development and characterization of salt tolerant somaclones in rice cultivar pokkali. Indian J Exp Biol.

[8] Chasse H, Boulben S, Costache V, Cormier P, Morales J (2017). Analysis of translation using polysome profiling. Nucleic Acids Res.

[9] Kawaguchi R, Girke T, Bray EA, Bailey-Serres J (2004). Differential mRNA translation contributes to gene regulation under non-stress and dehydration stress conditions in Arabidopsis thaliana. Plant J.

[10] Cohen A, Moses MS, Plant AL, Bray EA (1999). Multiple mechanisms control the expression of abscisic acid (ABA)-requiring genes in tomato plants exposed to soil water deficit. Plant Cell and Environment.

[11] DeRocher EJ, Bohnert HJ (1993). Development and environmental stress employ different mechanisms in the expression of a plant gene family. The Plant Cell Online.

[12] Jackson RJ, Hellen CU, Pestova TV. The mechanism of eukaryotic translation initiation and principles of its regulation. Nat Rev Mol Cell Biol. 2010;11(2):113–27. 10.1038/nrm2838 PMC4461372 20094052

[13] Haider S, Pal R (2013). Integrated analysis of transcriptomic and proteomic data. Curr Genomics.

[14] Jiao Y, Meyerowitz EM (2010). Cell-type specific analysis of translating RNAs in developing flowers reveals new levels of control. Mol Syst Biolvol.

[15] Moeller JR, Moscou MJ, Bancroft T, Skadsen RW, Wise RP, Whitham SA (2012). Differential accumulation of host mRNAs on polyribosomes during obligate pathogen-plant interactions. Mol BioSyst.

[16] Ingolia NT, Ghaemmaghami S, Newman JRS, Weissman JS (2009). Genome-wide analysis in vivo of translation with nucleotide resolution using ribosome profiling. Science.

[17] Wang T, Cui YZ, Jin JJ, Guo JH, Wang GB, Yin XF, He QY, Zhang G (2013). Translating mRNAs strongly correlate to proteins in a multivariate manner and their translation ratios are phenotype specific. Nucleic Acids Res.

[18] Kuersten S, Radek A, Vogel C, Penalva LO (2013). Translation regulation gets its ‘omics’ moment. Wiley Interdiscip Rev RNA.

[19] Ren Z-H, Gao J-P, Li L-G, Cai X-L, Huang W, Chao D-Y, Zhu M-Z, Wang Z-Y, Luan S, Lin H-X (2005). A rice quantitative trait locus for salt tolerance encodes a sodium transporter. Nat Genet.

[20] Cotsaftis O, Plett D, Johnson AAT, Walia H, Wilson C, Ismail AM, Close TJ, Tester M, Baumann U (2011). Root-specific transcript profiling of contrasting Rice genotypes in response to salinity stress. Mol Plant.

[21] Walia H, Wilson C, Condamine P, Liu X, Ismail AM, Zeng L, Wanamaker SI, Mandal J, Xu J, Cui X (2005). Comparative transcriptional profiling of two contrasting Rice genotypes under salinity stress during the vegetative growth stage. Plant Physiol.

[22] Walia H, Wilson C, Zeng L, Ismail A, Condamine P, Close T (2007). Genome-wide transcriptional analysis of salinity stressed japonica and indica rice genotypes during panicle initiation stage. Plant Mol Biol.

[23] Sturn A, Quackenbush J, Trajanoski Z (2002). Genesis: cluster analysis of microarray data. Bioinformatics.

[24] Puckette M, Iyer NJ, Tang Y, Dai XB, Zhao P, Mahalingam R (2012). Differential mRNA translation in Medicago truncatula accessions with contrasting responses to ozone-induced oxidative stress. Mol Plant.

[25] Kimura S, Higashino Y, Kitao Y, Masuda T, Urade R (2015). Expression and characterization of protein disulfide isomerase family proteins in bread wheat. BMC Plant Biol.

[26] Kong F, Mao S, Du K, Wu M, Zhou X, Chu C, Wang Y (2011). Comparative proteomics analysis of OsNAS1 transgenic Brassica napus under salt stress. Chin Sci Bull.

[27] Stec B (2006). Plant thionins - the structural perspective. Cell Mol Life Sci.

[28] Lakra N, Kaur C, Anwar K, Singla-Pareek SL, Pareek A (2018). Proteomics of contrasting rice genotypes: identification of potential targets for raising crops for saline environment. Plant Cell Environ.

[29] Gillis J, Pavlidis P (2012). “Guilt by association” is the exception rather than the rule in gene networks. PLoS Comput Biol.

[30] Kawasaki S, Borchert C, Deyholos M, Wang H, Brazille S, Kawai K, Galbraith D, Bohnert HJ (2001). Gene expression profiles during the initial phase of salt stress in Rice. The Plant Cell Online.

[31] Zörb C, Herbst R, Forreiter C, Schubert S (2009). Short-term effects of salt exposure on the maize chloroplast protein pattern. Proteomics.

[32] Le Gall H, Philippe F, Domon JM, Gillet F, Pelloux J, Rayon C (2015). Cell Wall metabolism in response to abiotic stress. Plants (Basel).

[33] Houston K, Tucker MR, Chowdhury J, Shirley N, Little A (2016). The plant Cell Wall: a complex and dynamic structure as revealed by the responses of genes under stress conditions. Front Plant Sci.

[34] Marowa P, Ding A, Kong Y (2016). Expansins: roles in plant growth and potential applications in crop improvement. Plant Cell Rep.

[35] Geilfus CM, Zorb C, Muhling KH (2010). Salt stress differentially affects growth-mediating beta-expansins in resistant and sensitive maize (Zea mays L.). Plant Physiol Biochem.

[36] Xu E, Chen M, He H, Zhan C, Cheng Y, Zhang H, Wang Z (2016). Proteomic analysis reveals proteins involved in seed imbibition under salt stress in Rice. Front Plant Sci.

[37] Senadheera P, Singh RK, Maathuis FJM (2009). Differentially expressed membrane transporters in rice roots may contribute to cultivar dependent salt tolerance. J Exp Bot.

[38] Sakurai J, Ishikawa F, Yamaguchi T, Uemura M, Maeshima M (2005). Identification of 33 Rice aquaporin genes and analysis of their expression and function. Plant Cell Physiol.

[39] Schmidt R, Schippers JHM, Welker A, Mieulet D, Guiderdoni E, Mueller-Roeber B (2012). Transcription factor OsHsfC1b regulates salt tolerance and development in Oryza sativa ssp. japonica. AoB Plants.

[40] Hanba YT, Shibasaka M, Hayashi Y, Hayakawa T, Kasamo K, Terashima I, Katsuhara M (2004). Overexpression of the barley aquaporin HvPIP2;1 increases internal CO2 conductance and CO2 assimilation in the leaves of transgenic Rice plants. Plant Cell Physiol.

[41] Mosa K, Kumar K, Chhikara S, McDermott J, Liu Z, Musante C, White J, Dhankher O (2012). Members of rice plasma membrane intrinsic proteins subfamily are involved in arsenite permeability and tolerance in plants. Transgenic Res.

[42] Panda S, Sahoo L, Katsuhara M, Matsumoto H (2013). Overexpression of alternative oxidase gene confers aluminum tolerance by altering the respiratory capacity and the response to oxidative stress in tobacco cells. Mol Biotechnol.

[43] Moons A (2003). Osgstu3 and osgtu4, encoding tau class glutathione S-transferases, are heavy metal- and hypoxic stress-induced and differentially salt stress-responsive in rice roots. FEBS Lett.

[44] Dixon DP, Skipsey M, Edwards R (2010). Roles for glutathione transferases in plant secondary metabolism. Phytochemistry.

[45] Kumar S, Asif M, Chakrabarty D, Tripathi R, Dubey R, Trivedi P (2013). Differential expression of Rice lambda class GST gene family members during plant growth, development, and in response to stress conditions. Plant Mol Biol Report.

[46] Sharma R, Sahoo A, Devendran R, Jain M (2014). Over-expression of a Rice tau class glutathione S-transferase gene improves tolerance to salinity and oxidative stresses in Arabidopsis. PLoS One.

[47] Kumar G, Kushwaha H, Panjabi-Sabharwal V, Kumari S, Joshi R, Karan R, Mittal S, Pareek SL, Pareek A (2012). Clustered metallothionein genes are co-regulated in rice and ectopic expression of OsMT1e-P confers multiple abiotic stress tolerance in tobacco via ROS scavenging. BMC Plant Biol.

[48] Deinlein U, Stephan AB, Horie T, Luo W, Xu G, Schroeder JI (2014). Plant salt-tolerance mechanisms. Trends Plant Sci.

[49] Tran L-SP, Nakashima K, Sakuma Y, Simpson SD, Fujita Y, Maruyama K, Fujita M, Seki M, Shinozaki K, Yamaguchi-Shinozaki K (2004). Isolation and functional analysis of Arabidopsis stress-inducible NAC transcription factors that bind to a drought-responsive cis-element in the early responsive to dehydration stress 1 promoter. The Plant Cell Online.

[50] Jiang Y, Deyholos M (2009). Functional characterization of Arabidopsis NaCl-inducible WRKY25 and WRKY33 transcription factors in abiotic stresses. Plant Mol Biol.

[51] Yang B, Jiang Y, Rahman M, Deyholos M, Kav N (2009). Identification and expression analysis of WRKY transcription factor genes in canola (Brassica napus L.) in response to fungal pathogens and hormone treatments. BMC Plant Biol.

[52] Razzaque S, Haque T, Elias SM, Rahman MS, Biswas S, Schwartz S, Ismail AM, Walia H, Juenger TE, Seraj ZI (2017). Reproductive stage physiological and transcriptional responses to salinity stress in reciprocal populations derived from tolerant (Horkuch) and susceptible (IR29) rice. Sci Rep.

[53] Bonilla P, Dvorak J, Mackill D, Deal K, Gregorio G. RFLP and SSLP mapping of salinity tolerance genes in chromosome 1 of rice (Oryza sativa L.) using recombinant inbred lines. Philippine Agricultural Scientist. 2002;65(1):68–76.

[54] Thomson M, de Ocampo M, Egdane J, Rahman MA, Sajise A, Adorada D, Tumimbang-Raiz E, Blumwald E, Seraj Z, Singh R (2010). Characterizing the Saltol quantitative trait locus for salinity tolerance in Rice. Rice.

[55] Alam R, Sazzadur Rahman M, Seraj ZI, Thomson MJ, Ismail AM, Tumimbang-Raiz E, Gregorio GB (2011). Investigation of seedling-stage salinity tolerance QTLs using backcross lines derived from Oryza sativa L. Pokkali. Plant Breed.

[56] Reddy AS (2007). Alternative splicing of pre-messenger RNAs in plants in the genomic era. Annu Rev Plant Biol.

[57] Park SY, Grabau E (2017). Bypassing miRNA-mediated gene regulation under drought stress: alternative splicing affects CSD1 gene expression. Plant Mol Biol.

[58] Filichkin SA, Priest HD, Givan SA, Shen R, Bryant DW, Fox SE, Wong WK, Mockler TC (2010). Genome-wide mapping of alternative splicing in Arabidopsis thaliana. Genome Res.

[59] Kong J, Gong J-M, Zhang Z-G, Zhang J-S, Chen S-Y (2003). A new AOX homologous gene OsIM1 from rice (Oryza sativa L.) with an alternative splicing mechanism under salt stress. Theor Appl Genet.

[60] Matsukura S, Mizoi J, Yoshida T, Todaka D, Ito Y, Maruyama K, Shinozaki K, Yamaguchi-Shinozaki K (2010). Comprehensive analysis of rice DREB2-type genes that encode transcription factors involved in the expression of abiotic stress-responsive genes. Mol Gen Genomics.

[61] Pitann B, Zörb C, Mühling KH (2009). Comparative proteome analysis of maize (Zea mays L.) expansins under salinity. J Plant Nutr Soil Sci.

[62] Kosova K, Vitamvas P, Prasil IT, Renaut J (2011). Plant proteome changes under abiotic stress--contribution of proteomics studies to understanding plant stress response. J Proteome.

[63] Gygi SP, Rochon Y, Franza BR, Aebersold R (1999). Correlation between protein and mRNA abundance in yeast. Mol Cell Biol.

[64] Bogeat-Triboulot MB, Brosche M, Renaut J, Jouve L, Le Thiec D, Fayyaz P, Vinocur B, Witters E, Laukens K, Teichmann T (2007). Gradual soil water depletion results in reversible changes of gene expression, protein profiles, ecophysiology, and growth performance in Populus euphratica, a poplar growing in arid regions. Plant Physiol.

[65] Yoshida S, Forno DA, Cock JH, Gomez KA. Laboratory manual for physiological studies of Rice. IRRI, Las Banos, Laguna. 1976;61–5.

[66] Li YF, Mahalingam R, Sunkar R (2017). Isolation of Polysomal RNA for analyzing stress-responsive genes regulated at the translational level in plants. Methods Mol Biol.

[67] Trapnell C, Williams BA, Pertea G, Mortazavi A, Kwan G, van Baren MJ, Salzberg SL, Wold BJ, Pachter L (2010). Transcript assembly and quantification by RNA-Seq reveals unannotated transcripts and isoform switching during cell differentiation. Nat Biotechnol.

[68] Trapnell C, Pachter L, Salzberg SL (2009). TopHat: discovering splice junctions with RNA-Seq. Bioinformatics.

[69] Li RQ, Yu C, Li YR, Lam TW, Yiu SM, Kristiansen K, Wang J (2009). SOAP2: an improved ultrafast tool for short read alignment. Bioinformatics.

[70] Tian T, Liu Y, Yan HY, You Q, Yi X, Du Z, Xu WY, Su Z (2017). agriGO v2.0: a GO analysis toolkit for the agricultural community, 2017 update. Nucleic Acids Res.

[71] Supek F, Bosnjak M, Skunca N, Smuc T (2011). REVIGO summarizes and visualizes long lists of gene ontology terms. PLoS One.

[72] Thimm O, Blasing O, Gibon Y, Nagel A, Meyer S, Kruger P, Selbig J, Muller LA, Rhee SY, Stitt M (2004). MAPMAN: a user-driven tool to display genomics data sets onto diagrams of metabolic pathways and other biological processes. Plant J.

