# Supplementary figures and images for: Comparative transcriptome and translatome analysis in contrasting rice genotypes reveals differential mRNA translation in salt-tolerant Pokkali under salt stress
Source: BMC Genomics. 2018 Dec 31;19(Suppl 10):935. doi: 10.1186/s12864-018-5279-4 (PMC6311934; doi:10.1186/s12864-018-5279-4)

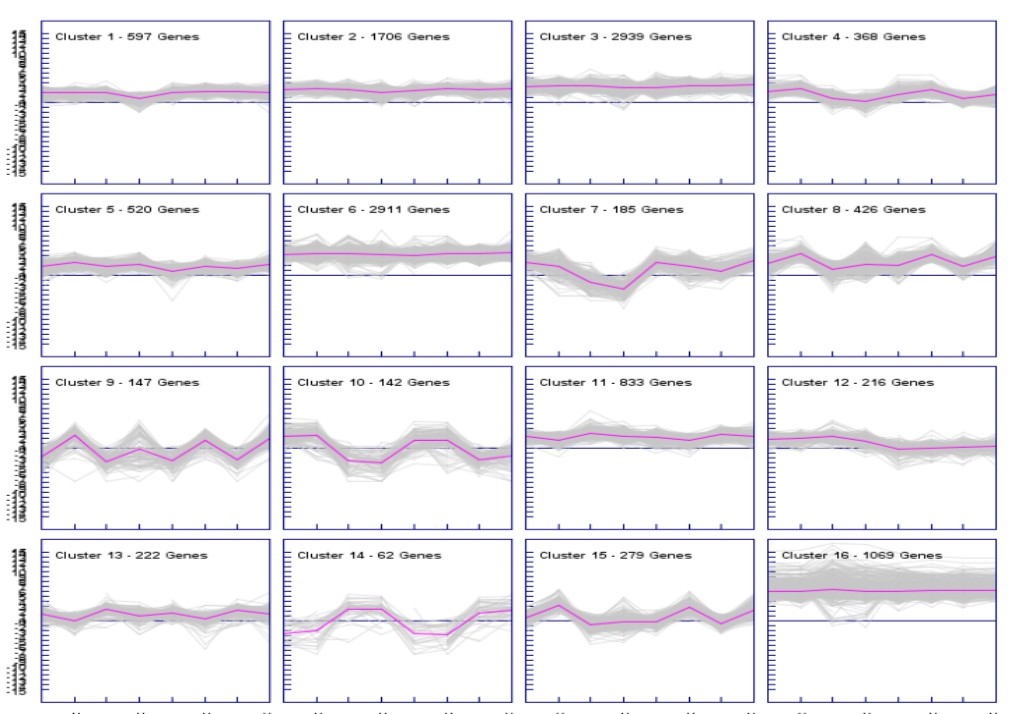

Supplement: Supplementary file 2 — Figure S1. Overview expression patterns of transcriptome and translatome of IR29 and Pok under control and salt stress conditions. The purple line represents the mean expression level of the genes in the same cluster; grey lines represent the expression of each gene in the cluster. Transcripts were divided into 16 clusters (cluster 1 to 16) based on their expression level by using k-Means Clustering (KMC). The vertical axis represents the gene expression value (log2 FPKM), and the horizontal axis represents the 8 samples (in order from left to right: IR29c_RNA, IR29s_RNA, Pokc_RNA, Poks_RNA, IR29c_PS, IR29s_PS, Pokc_PS and Poks_PS).Most genes in clusters 1, 2, 3, 6 and 16 had similar expression in IR29 and Pok but were not responsive by salt stress. Cluster 5 represents genes that were moderately upregulated by salt stress, cluster 9 represents genes that were strongly upregulated by salt stress in both IR29 and Pok at the transcriptional and translational levels, and cluster 13 represents genes were repressed by salt stress in IR29 and Pok at both transcriptional and translational levels. Cluster 10 represents genes with higher abundances at both transcriptional and translational levels in IR29 but extremely low abundance in Pok, whereas cluster 14 represents genes with very low expression at both transcriptional and translational levels in IR29 under both control and salt stress conditions but higher transcriptional and translational levels in Pok; these genes did not respond to salt stress. Cluster 7 represents genes that did not respond to salt stress at the transcriptional level in Pok but were highly upregulated at the translational level. Clusters 8 and 15 represent genes highly upregulated by salt stress in IR29 at the transcriptional and translational levels but only upregulated in Pok at the translational level. Cluster 11 represents genes with relatively higher expression in Pok than in IR29 at the transcriptional level. Cluster 12 represents genes with l [file 12864_2018_5279_MOESM2_ESM.jpg]

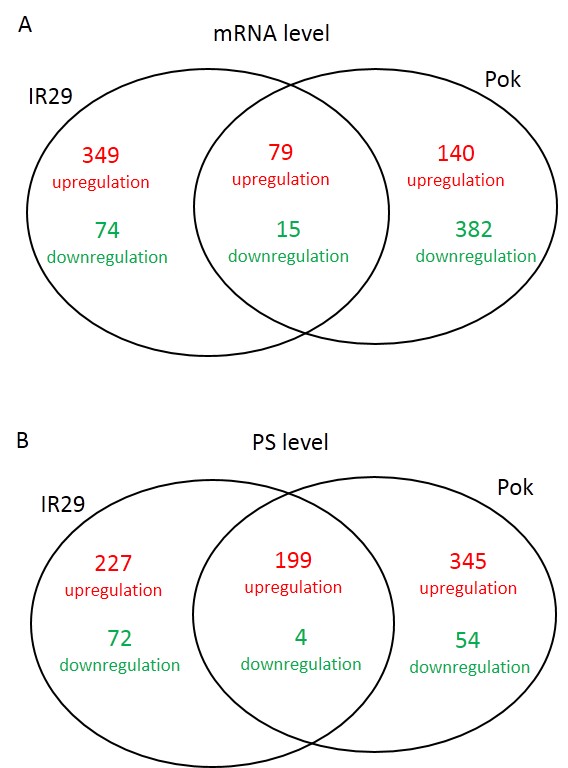

Supplement: Supplementary file 6 — Figure S2. Comparative analysis of DEGs between IR29 and Pok at the transcriptional level (A) and translational level B). (JPG 65 kb) [file 12864_2018_5279_MOESM6_ESM.jpg]

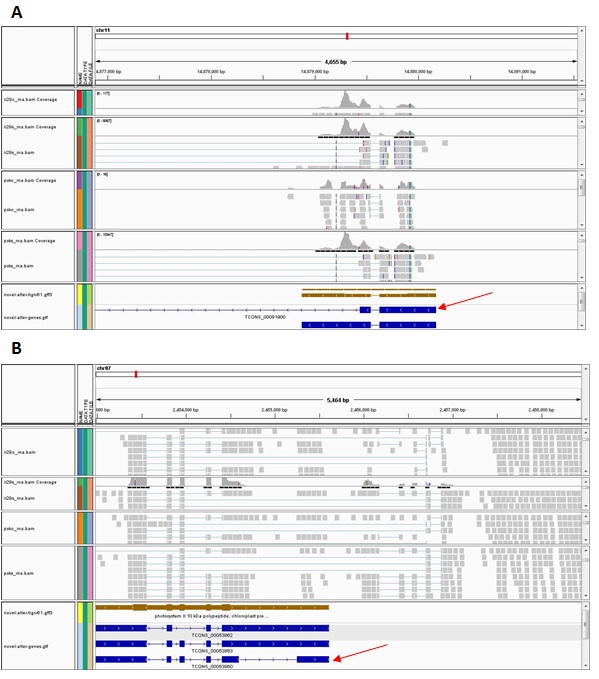

Supplement: Supplementary file 15 — Figure S3. Novel alternate splice variants of dehydrin (Os11g26790) (A) and photosystem II 10-kDa polypeptide, chloroplast precursor (Os07g05360) (B). (JPG 112 kb) [file 12864_2018_5279_MOESM15_ESM.jpg]
